# GPR55 is expressed in glutamate neurons and functionally modulates drug taking and seeking in rats and mice

**DOI:** 10.1038/s41398-024-02820-3

**Published:** 2024-02-19

**Authors:** Yi He, Hui Shen, Guo-Hua Bi, Hai-Ying Zhang, Omar Soler-Cedeño, Hannah Alton, Yihong Yang, Zheng-Xiong Xi

**Affiliations:** 1 Addiction Biology Unit, Molecular Targets and Medications Discovery Branch, National Institute on Drug Abuse, Intramural Research Program, Baltimore, MD 21224 USA; 2 Neuroimaging Research Branch, Intramural Research Program, National Institute on Drug Abuse, Baltimore, MD 21224 USA; 3 Medication Development Program, National Institute on Drug Abuse, Intramural Research Program, Baltimore, MD 21224 USA; 4 Postdoctoral Research Associate Training Fellow, National Institute of General Medical Sciences, Bethesda, MD 20892 USA

**Keywords:** Molecular neuroscience, Pharmacodynamics

## Abstract

G protein-coupled receptor 55 (GPR55) has been thought to be a putative cannabinoid receptor. However, little is known about its functional role in cannabinoid action and substance use disorders. Here we report that GPR55 is predominantly found in glutamate neurons in the brain, and its activation reduces self-administration of cocaine and nicotine in rats and mice. Using RNAscope in situ hybridization, GPR55 mRNA was identified in cortical vesicular glutamate transporter 1 (VgluT1)-positive and subcortical VgluT2-positive glutamate neurons, with no detection in midbrain dopamine (DA) neurons. Immunohistochemistry detected a GPR55-like signal in both wildtype and GPR55-knockout mice, suggesting non-specific staining. However, analysis using a fluorescent CB1/GPR55 ligand (T1117) in CB1-knockout mice confirmed GPR55 binding in glutamate neurons, not in midbrain DA neurons. Systemic administration of the GPR55 agonist O-1602 didnt impact ∆^9^-THC-induced analgesia, hypothermia and catalepsy, but significantly mitigated cocaine-enhanced brain-stimulation reward caused by optogenetic activation of midbrain DA neurons. O-1602 alone failed to alter extracellar DA, but elevated extracellular glutamate, in the nucleus accumbens. In addition, O-1602 also demonstrated inhibitory effects on cocaine or nicotine self-administration under low fixed-ratio and/or progressive-ratio reinforcement schedules in rats and wildtype mice, with no such effects observed in GPR55-knockout mice. Together, these findings suggest that GPR55 activation may functionally modulate drug-taking and drug-seeking behavior possibly via a glutamate-dependent mechanism, and therefore, GPR55 deserves further study as a new therapeutic target for treating substance use disorders.

The endocannabinoid system is implicated in numerous physiological functions, such as emotion, locomotion, cognition, learning and memory, as well as cannabis action and drugs of abuse [1–5]. Extensive research in the past several decades indicates that cannabinoid CB1 and CB2 receptors are the major receptors in the brain involved in the CNS effects of cannabinoids [5, 6]. However, cannabinoids also bind to other non-CB1 and non-CB2 receptors, such as G protein-coupled receptor 55 (GPR55) [5, 7], while little is known about the functional role of GPR55 in cannabinoid action and other substance use disorders.

GPR55 was first isolated in 1999 as an orphan GPCR with high expression levels in the human striatum [8]. Initially, it was identified as a potential purinergic or chemokine-like receptor based on amino acid homology [8]. However, further studies indicate that GPR55 could be another putative cannabinoid receptor because multiple cannabinoids, including the endocannabinoids (anandamide and 2-arachidonoylglycerol), phytocannabinoid (Δ^9^-tetrahydrocannabinol, ∆^9^-THC), and synthetic cannabinoids (HU-210, CP55,940), can bind and activate GPR55 [7, 9]. Lysophosphatidylinositol (LPI) has been identified as an endogenous ligand of GPR55 [10, 11]. Structurally, GPR55 is distinct from both CB1 and CB2 receptors because it lacks the classical endocannabinoid binding pocket [12] and has minimal receptor homology with CB1 (13.5%) or CB2 receptors (14.4%) [13].

Quantitative RT-PCR assays revealed GPR55 gene expression in the mouse brain, including the frontal cortex, striatum, hippocampus, and cerebellum [9, 14]. In situ hybridization (ISH) and immunohistochemistry (IHC) assays also show GPR55 mRNA and immunostaining signals in neurons in the striatum and hippocampus [15–18]. However, the detected GPR55 signals are very weak, and the phenotypes of neurons that express GPR55 in the striatum and hippocampus are largely unknown. It is also unclear whether functional GPR55 is expressed on cortical or subcortical glutamate neurons and midbrain DA neurons.

Functionally, GPR55 has been shown to modulate anxiety-related behavior [19, 20], pain perception [21], locomotion [14, 22], learning and memory [23], and the conditioned place preference (CPP) response to morphine or nicotine [24–26]. In our recent study, we demonstrated that the genetic deletion of CB1 or CB2 receptors led to a reduction, whereas the genetic deletion or pharmacological blockade of GPR55 enhanced behavioral responses to cannabinoids such as Δ^9^-THC and WIN55,212-2 in analgesia, hypothermia, and catalepsy tests [22]. These findings suggest that CB1/CB2 and GPR55 play opposing roles in mediating classical cannabinoid effects. Consequently, we proposed that GPR55 agonists, rather than antagonists, might hold therapeutic potential in reducing cannabinoid actions mediated by activation of CB1 and CB2 receptors or in reducing action produced by other substances of abuse. However, direct supporting evidence is lacking.

In this study, we first used advanced RNAscope ISH, IHC, and fluorescent ligand binding assays to examine the GPR55 gene/receptor expression in brain DA and glutamate neurons. We then examined whether systemic administration of O-1602, a potent GPR55 agonist (with EC_50_ values of 13, >30,000, and >30,000 nM for GPR55, CB1 and CB2 receptors, respectively) [9], alters DA or glutamate release in the nucleus accumbens (NAc) in rats using in vivo microdialysis. Next, we examined the effects of O-1602 pretreatment on ∆^9^-THC-induced triad effects (analgesia, hypothermia and catalepsy), DA-dependent optical intracranial self-stimulation (oICSS), cocaine effects on oICSS, and intravenous cocaine or nicotine self-administration in rats and mice. Lastly, we examined the effects of O-1602 on oral sucrose self-administration and open-field locomotion to determine whether O-1602 selectively alter drug-taking and drug-seeking behavior. GPR55-knockout (GPR55-KO) mice were used as negative controls to determine GPR55 signal specificity and O-1602 pharmacological specificity. We found that GPR55 is mainly expressed in glutamate neurons in the brain and functionally modulates cocaine and nicotine self-administration.

## Methods and materials

Male adult Long-Evans and alcohol-preferring rats were used in the present study. Male and female WT, CB1-KO and GPR55-KO mice were bred in the animal facility of the National Institute on Drug Abuse Intramural Research Program. The full descriptions of the experimental animals, the experimental methods, and the data analysis are provided in Supplementary Information.

### Experiment 1: RNAscope ISH

We first performed RNAscope ISH to examine the cellular distribution of GPR55 mRNA in cortical and subcortical glutamate neurons as well as midbrain DA neurons. The complete RNAscope procedures are described in *Supplementary Information*.

### Experiment 2: Immunohistochemistry (IHC)

We then used double-label IHC to examine GPR55 immunostaining in WT and GPR55-KO mice. The complete IHC procedures are described in Supplementary Information.

### Experiment 3: Fluorescent GPR55 ligand binding assays

Due to concerns about the GPR55 antibody specificity, we next used a fluorescent CB1/GPR55 ligand (Tocrifluor T1117) to examine T1117 binding on GPR55 [27, 28] in CB1-KO mice. CB1-KO mice were used in this assay because T1117 is a fluorescent analog of AM251 (a selective CB1 receptor antagonist) and has been shown to have low binding affinity to CB1 receptor [27, 28], thus the use of CB1-KO mice will exclude its possible binding to CB1 receptor. The complete procedures for T1117 binding are provided in Supplmentary Information.

### Experiment 4: Optical intracranial self-stimulation (oICSS)

This experiment was designed to determine whether the GPR55 agonist O-1602 alters DA-dependent oICSS or cocaine action in oICSS. The complete oICSS procedures are described in Supplementary Information.

### Experiment 5: In vivo microdialysis in P-rats

This experiment was to determine whether O-1602 alters DA or glutamate release in the NAc. The complete microdialysis procedures are described in Supplementary Information.

### Experiment 6: ∆^9^-THC-induced triad effects

This experiment was to determine whether activation of GPR55 by O-1602 alters ∆^9^-THC-induced classical triad effects—analgesia, hypothermia and catalepsy. The complete triad experimental procedures are described in Supplementary Information.

### Experiment 7: Intravenous drug self-administration in rats and mice

This experiment was to determine whether O-1602 is able to inhibit intravenous cocaine or nicotine self-administration by activation of GPR55. The complete drug self-administration procedures are described in Supplementary Information.

### Experiment 8: Oral sucrose self-administration and open-field locomotion

The complete procedures are described in Supplementary Information.

### Data analysis

Data analyses and graphing were accomplished using SigmaPlot software. One-way or two-way repeated measures (RM) ANOVAs were used for evaluating the effects of treatment on drug or sucrose self-administration, locomotion, extracellular DA/glutamate, and oICSS behavior. Graphs were made based on the results reported as mean ± SEM. *p* < 0.05 was defined as a statistically significant difference.

## Results

### GPR55 mRNA expression in glutamate, not dopamine, neurons

**Fig. 1:**
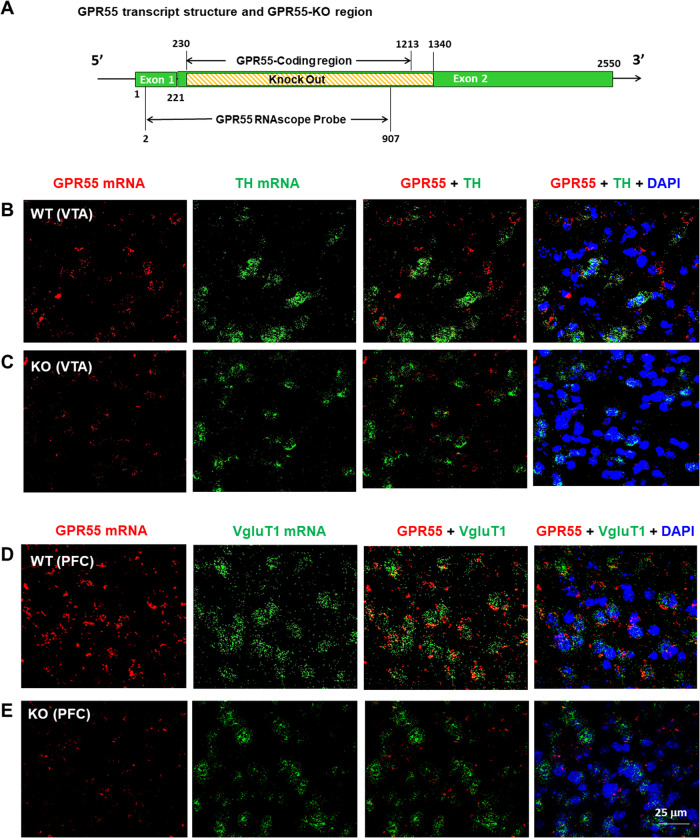
GPR55 RNAscope ISH results in WT and GPR55-KO mice. **A** Diagram showing the gene knockout strategy for generating GPR55-KO mice, in which the GPR55-coding gene sequence located on Exon 2 was fully deleted. **B**, **C** GPR55 mRNA was detected in the VTA in WT mice (**B**), but not in GPR55-KO mice (**C**). However, GPR55 did not show colocalization with TH mRNA, a DA neuronal marker, in the VTA of WT mice (**B**). **D**, **E** High-density GPR55 mRNA was also detected in the PFC of WT mice (**D**), but not in GPR55-KO mice (**E**). Notably, GPR55 mRNA colocalizes with VgluT1 mRNA, a cortical glutamatergic neuronal marker, in the PFC of WT mice (**D**). (Also see Figs. S1, 2).

To determine the cellular distributions of GPR55 in the brain, we first used RNAscope ISH assays to examine GPR55 transcript (mRNA) expression in brain DA neurons and glutamate neurons. Figure 1A depicts the GPR55 gene structure and gene targeting strategy, illustrating that a portion of exon 2 of the gpr55 gene, which includes the entire coding region of the GPR55 protein, is deleted in the utilized GPR55-KO (null mutant) mice [29]. This strain of GPR55-KO mice exhibited normal brain development, synaptic transmission, body weight, gross motor movement, and learning behaviors, except mildly impaired movement coordination [14].

RNAscope in situ hybridization assays detected significant GPR55 mRNA staining in WT (Fig. 1B, D), not GPR55-KO (Fig. 1C, E), mice in either the ventral tegmental area (VTA) (Fig. 1B, C) of the midbrain or the prefrontal cortex (PFC) (Fig. 1D, E). Notably, GPR55 mRNA is not colocalized with tyrosine hydroxylase (TH) in DA neurons in (VTA) of the midbrain (Fig. 1B), but it is colocalized with vesicular glutamate transporter 1 (VgluT1) in cortical glutamate neurons in the prefrontal cortex (PFC) (Fig. 1D). We also examined colocalization of GPR55 and DA transporter (DAT, another DA neuronal marker) mRNAs in the VTA, but failed to detect their colocalization (Fig. S1). Figure S2 shows a high-magnification image from Fig. 1D, illustrating clear GPR55 mRNA and VgluT1 mRNA colocalization in cortical glutamate neurons. Quantitative cell counting assays indicated that ~90% (91.65 ± 12.43%) of cortical glutamate neurons express GPR55 in WT mice, while only ~10% (10.42 ± 2.14%) of glutamate neurons show faint GPR55-like signal in the GPR55-KO mice. We also examined GPR55 mRNA expression in other brain regions. We detected similar GPR55 and VgluT2 colocalization in the hippocampus and thalamus, but not in the striatum (Fig. S3).

### GPR55-immunostaining is not highly GPR55-specific

Next, we used IHC assays to detect GPR55 protein expression in the regions where mRNA was detected. We used two commercially available GPR55 antibodies: a polyclonal anti-GPR55 antibody (Abcam), which targets the C-terminus of human GPR55, and another polyclonal anti-GPR55 antibody with an undisclosed epitope (Cayman Chemicals). Figures S4 and S5 show the representative GPR55-immunostaining images, illustrating that GPR55-like signal was detected in the VTA, but it was not colocalized with TH-immunostaining in DA neurons when using either the Abcam GPR55 antibody (Fig. S4) or the Cayman GPR55 antibody (Fig. S5). However, the GPR55-immunostaining appears not highly specific as it is still detectable in either GPR55-KO mice (Fig. S4B) or in the presence of either of the GPR55 antibody immune peptides (Figs. S4C and S5B). These findings suggest that both the antibodies against the human or bovine GPR55 are not suitable to detect GPR55 receptor proteins in mice, although human GPR55 show 75% and 78% homology with the rat and mouse GPR55 proteins, respectively [9].

### Fluorescent cannabinoid ligand binding reveals GPR55 expression in glutamate, not DA, neurons

**Fig. 2:**
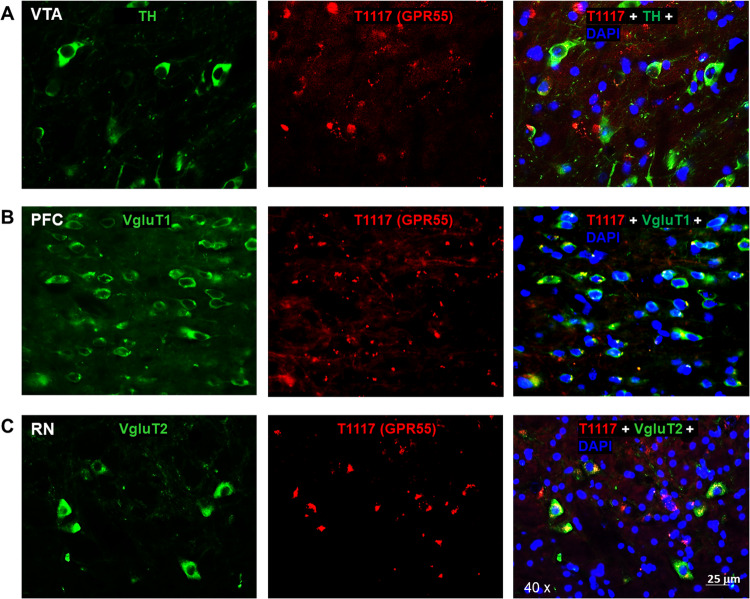
T1117 fluorescent ligand binding results in CB1-KO mice. T1117 binding signal is not colocalized with TH-immunostaining in VTA DA neurons (**A**), but it is colocalized with VgluT1- or VgluT2-immunostaining in glutamate neurons in the PFC) (**B**) and red nucleus (RN) (**C**).

**Fig. 3:**
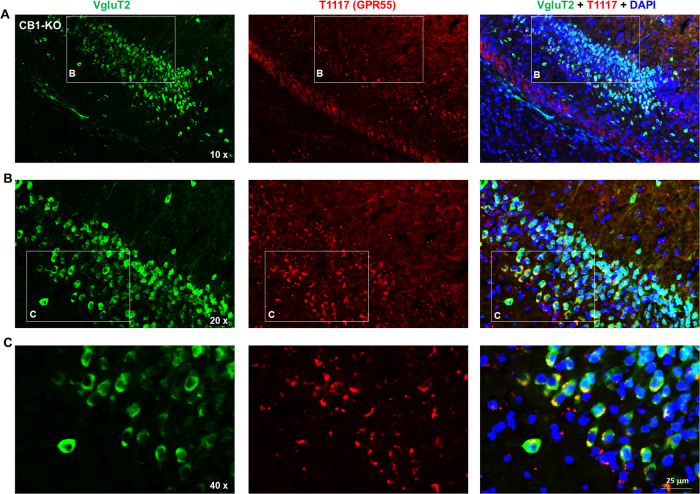
Fluorescent ligand (T1117) binding results in the hippocampus of CB1-KO mice. T1117-labeled GPR55 is colocalized with VgluT2-immunostaining in hippocampal glutamate neurons of CB1-KO mice. The images were taken under different magnifications from 10X (**A**) to 20X (**B**) and 40X (**C**).

To further validate our findings by RNAscope ISH assays, we then used a fluorescent cannabinoid ligand - Tocrifluor T1117 (T1117) to detect GPR55 expression in the brain. As T1117 is an analog of AM251, a selective CB1 receptor antagonist, and has been shown to have low binding affinity to the CB1 receptor at high concentrations [27, 28], we used CB1-KO mice to exclude its binding to the CB1 receptor in this assay. There are two types of glutamate neurons that express VgluT1 mainly in the cortex and VgluT2 mainly in the subcortical brain regions such as the hippocampus and thalamus [30]. Therefore, we used two different glutamatergic neuronal markers (e.g., VgluT1 and VgluT2 antibodies) to identify glutamate neurons in this study. Figure 2 shows representative T1117 binding in the VTA, PFC, and midbrain red nucleus (RN), illustrating that T1117 did not show colocalization with TH in VTA DA neurons (Fig. 2A), but showed clear T1117-VgluT1 colocalization in PFC glutamate neurons (Fig. 2B) and T1117-VgluT2 colocalization in RN glutamate neurons (Fig. 2C). Notably, T1117 fluorescent signal is also detected in other non-DA neurons in the VTA (Fig. 2A) or non-glutamate neurons in the RN (Fig. 2C). Figure 3 shows the representative T1117 binding images in the hippocampus under different magnifications (×10, ×20, and ×40), indicating that T1117 and VgluT2 colocalization in the majority of VgluT2-positive glutamate neurons in the hippocampus of CB1-KO mice. We did not use GPR55-KO mice to determine the signal specificity as T1117 also binds to CB1 receptor in GPR55-KO mice and double CB1-KO and GPR55-KO mice are currently not available. Together, these fluorescent ligand binding data, combined with our data from RNAscope ISH and IHC assays, support a conclusion that GPR55 is mainly expressed in cortical and subcortical glutamate neurons, but it is not expressed in midbrain DA neurons.

### O-1602 failed to alter ∆^9^-THC-induced triad effects

As stated above, genetic deletion or pharmacological blockade of GPR55 produced an enhanced behavioral response to ∆^9^-THC in hot-plate analgesia, hypothermia, and catalepsy [22], suggesting that GPR55 agonists may have therapeutic effects against cannabinoid action mediated by activation of CB1 and CB2 receptors. To test this hypothesis, we first observed the effects of O-1602 (a potent GPR55 agonist) on ∆^9^-THC-induced classical triad effects. We found treatment with O-1602 (10, 20 mg/kg, i.p., 15 min prior to ∆^9^-THC) failed to alter 30 mg/kg ∆^9^-THC-induced analgesia, hypothermia, or catalepsy (Fig. S6). Two-way RM ANOVA did not reveal a significant O-1602 treatment main effect in ∆^9^-THC-induced analgesia (Fig. S6A, *F*_2,14_ = 0.994, *p* > 0.05), ∆^9^-THC-induced hypothermia (Fig. S6B, *F* = 0.441, *p* > 0.05), or ∆^9^-THC-induced catalepsy (Fig. S6C, *F*_2,14_ = 2.31, *p* > 0.05). These findings suggest that O-1602, at the tested doses, is unable to counteract the action produced by ∆^9^-THC in these behavioral assays.

### O-1602 itself failed to alter DA-dependent oICSS

**Fig. 4:**
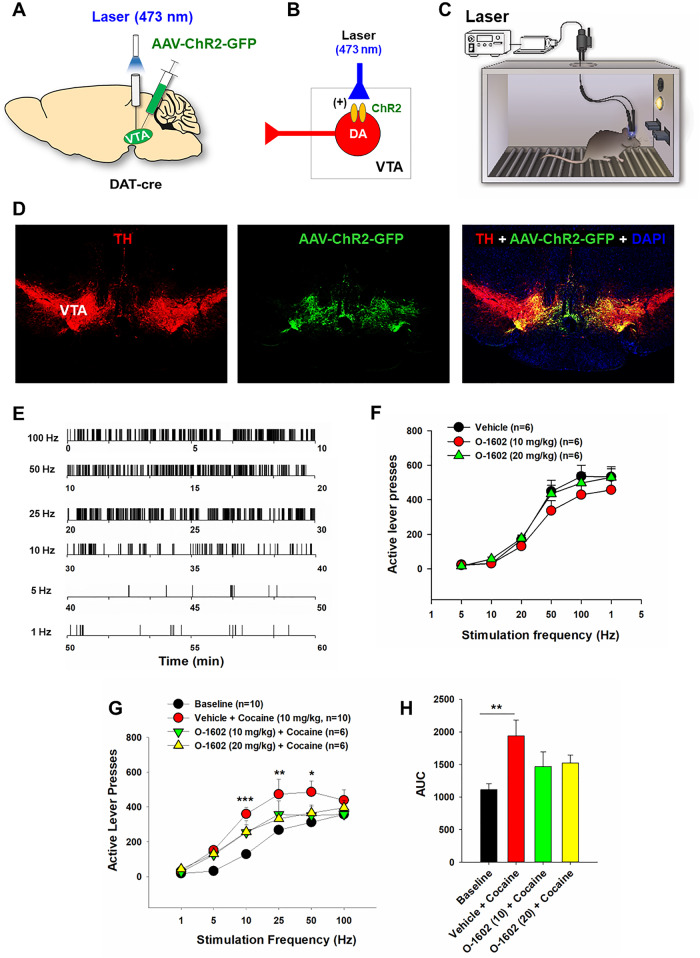
Effects of O-1602 on DA-dependent optical intracranial self-stimulation (oICSS) in DAT-Cre mice. **A** A schematic diagram of the AAV-ChR2-GFP microinjection and intracranial optical fiber implantation within the VTA. **B** A diagram showing AAV-ChR2-GFP expression in VTA DA neurons, which can be activated by 473 nm laser stimulation. **C** A diagram of the setup of the oICSS experiment. **D** Representative immunostaining images in a midbrain slice, indicating the colocalization of AAV-ChR2-GFP and TH in the VTA DA neurons. **E** Representative lever responding to different frequencies of laser stimulation in a single session from a single mouse. **F** Systemic administration of O-1602 failed to significantly alter DA-dependent oICSS behavior. **G**, **H** Systemic administration of O-1602 dose-dependently attenuated cocaine-enhanced oICSS in DAT-Cre mice.

Given the important role of DA in cannabinoid action and drug abuse, we then examined the effects of O-1602 on DA-dependent optical intracranial self-stimulation (oICSS) behavior in DAT-Cre mice. In this experiment, AAV-ChR2-GFP vectors were microinjected into bilateral VTA to express light-sensitive ChR2 proteins in midbrain DA neurons (Fig. 4A, B), and then the mice were trained to press an active lever to deliver laser to self-stimulate VTA DA neurons (Fig. 4C, D). In consistent with our previous reports [31, 32], optical stimulation of VTA DA neurons is reinforcing as assessed by robust oICSS responses in a stimulation frequency-dependent manner (Fig. 4E). Systemic administration of O-1602, at the doses of 10 and 20 mg/kg, did not significantly alter oICSS in DAT-Cre mice (Fig. 4F), suggesting that O-1602 is not reward-enhancing or reward-attenuating by itself. This is consistent with our imaging findings that GPR55 genes and proteins are not identified in midbrain DA neurons (Figs. 1 and 2; Figs. S4 and S5).

### O-1602 attenuated cocaine-enhanced oICSS

We have previously reported that drugs of abuse such as cocaine and oxycodone produce enhancement in oICSS in DAT-Cre mice [31, 32]. In this study, we investigated whether pretreatment with O-1602 could alter cocaine-enhanced oICSS. Indeed, cocaine, at 10 mg/kg, significantly enhanced oICSS and shifted the frequency-rate response curve upward (Fig. 4G). Pretreatment with O-1602, at 10 or 20 mg/kg, significantly mitigated cocaine-enhanced oICSS (Fig. 4G). Two-way RM ANOVA revealed a significant drug treatment main effect (*F*_3,27_ = 4.05, *p* < 0.05), stimulation frequency main effect (*F*_5,131_ = 78.83, *p* < 0.001) and treatment × frequency interaction (F_15, 131_ = 1.99, *p* < 0.05). Post-hoc individual group comparisons reveal significant differences only between the baseline and (vehicle + cocaine) groups at 10, 25, and 50 Hz, but not between the baseline and any of the (O-1602 + cocaine) groups. A one-way ANOVA for the area under the curve (AUC) data also revealed a significant drug treatment main effect (Fig. 4H, *F*_3,29_ = 4.58, *p* = 0.01). Post-hoc group comparisons revealed a significant difference only between the baseline and (vehicle + cocaine) groups, not between other groups.

### O-1602 elevates NAc extracellular glutamate, not DA, in rats

**Fig. 5:**
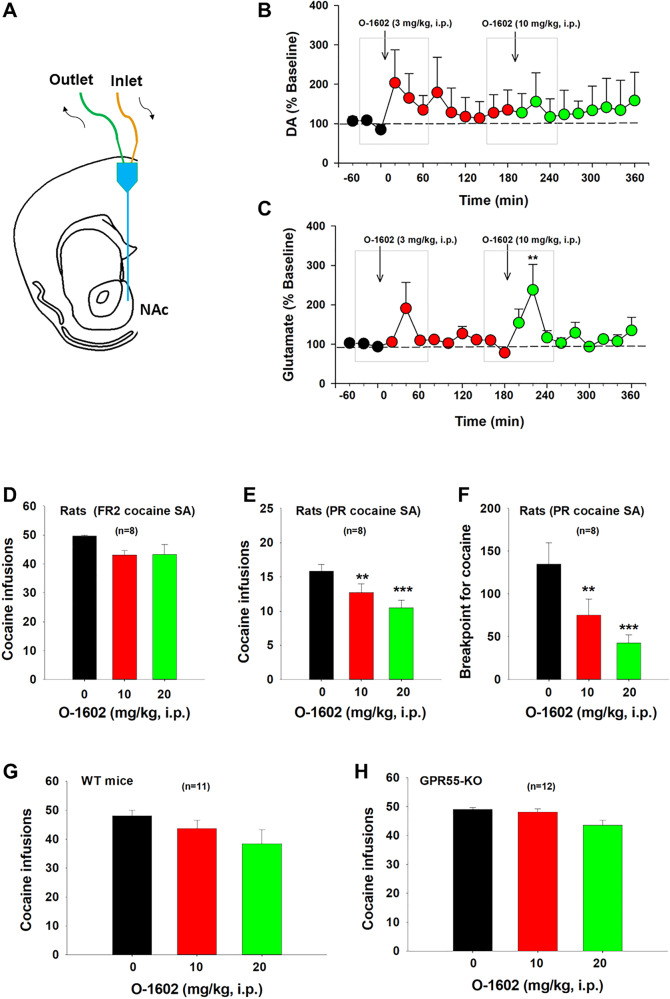
Effects of O-1602 on extracellular DA and glutamate levels in the NAc and cocaine self-administration (SA) in rats and mice. **A** A diagram showing a microdialysis probe targeting the NAc-shell. **B**, **C** Systemic administration of O-1602 failed to alter extracellular DA in the NAc (**B**), while it dose-dependently elevated extracellular glutamate levels in the NAc (**C**) in rats after O-1602 administration. **D** Systemic administration of O-1602 failed to alter cocaine SA under FR2 reinstatement schedule in rats. **E**, **F** Systemic administration of O-1602 dose-dependently inhibited cocaine SA under PR reinforcement schedule as assessed by the number of cocaine infusions (**E**) or breakpoint level for cocaine self-administration (**F**). **G**, **H** Systemic administration of O-1602 did not produce a significant reduction in cocaine SA in WT or GPR55-KO mice under FR1 reinforcement schedule. **p < 0.01, ***p < 0.001, com*p*ared to the vehicle control group.

Next, we used in vivo brain microdialysis (Fig. 5A) to examine whether activation of GPR55 alters glutamate or DA release in the NAc, a critical brain region involved in drug abuse [2, 5]. We selected alcohol-preferring rats (P-rats) for this experiment based on our behavioral assays, which demonstrated that O-1602 displayed higher efficacy in inhibiting nicotine self-administration in P-rats. We found that O-1602, at 3 and 10 mg/kg, failed to alter extracellular DA (Fig. 5B), but produced a significant increase in extracellular glutamate levels in the NAc in a dose-dependent manner (Fig. 5C). An one-way RM ANOVA for the data, shown in the gray boxes, did not reveal an O-1602 treatment main effect on extracellular DA after 3 mg/kg (Fig. 5B, *F*_4,20_ = 1.16, *p* > 0.05) or 10 mg/kg (Fig. 5B, *F*_4,20_ = 0.51, *p* > 0.05) O-1602 administration. However, the one-way RM ANOVA revealed a significant O-1602 treatment main effect in extracellular glutamate after 10 mg/kg (Fig. 5C, *F*_2,24_ = 4.17, *p* = 0.01), but not after 3 mg/kg (Fig. 5C, *F*_4,24_ = 1.76, *p* > 0.05), O-1602 administration.

### O-1602 inhibits cocaine self-administration under progressive-ratio schedule in rats

We have recently reported that the elevation of extracellular glutamate in the NAc by blockade of glial GLT-1 inhibits cocaine self-administration [33]. Next, we examined whether O-1602, which also elevates extracellular glutamate in the NAc (Fig. 5C), is able to inhibit intravenous cocaine self-administration in rats and mice. Systemic administration of O-1602, at 10 mg/kg and 20 mg/kg, failed to alter cocaine self-administration under FR2 reinforcement in rats (Fig. 5D, one-way RM ANOVA did not reveal significant O-1602 treatment main effects (*F*_2,14_ = 3.69, *p* > 0.05). However, under progressive-ratio reinforcement schedules, the same doses of O-1602 dose-dependently inhibited cocaine self-administration as assessed by the number of cocaine infusions (Fig. 5E, *F*_2,14_ = 13.43, *p* < 0.001) or breakpoint level for cocaine self-administration (Fig. 5F, *F*_2, 14_ = 10.98, *p* = 0.001).

We also observed the effects of O-1602 on cocaine self-administration in both WT and GPR55-KO mice. We found that O-1602 failed to significantly inhibit cocaine self-administration under FR1 reinforcement in wildtype mice (Fig. 5G, *F*_2,20_ = 1.40, *p* > 0.05) or GPR55-KO mice Fig. 5H, *F*_2,22_ = 1.18, *p* > 0.05), suggesting that O-1602 is unable to inhibit cocaine use when cocaine is readily available under low effort (FR1, FR2) experimental conditions.

### O-1602 inhibits nicotine self-administration in rats

**Fig. 6:**
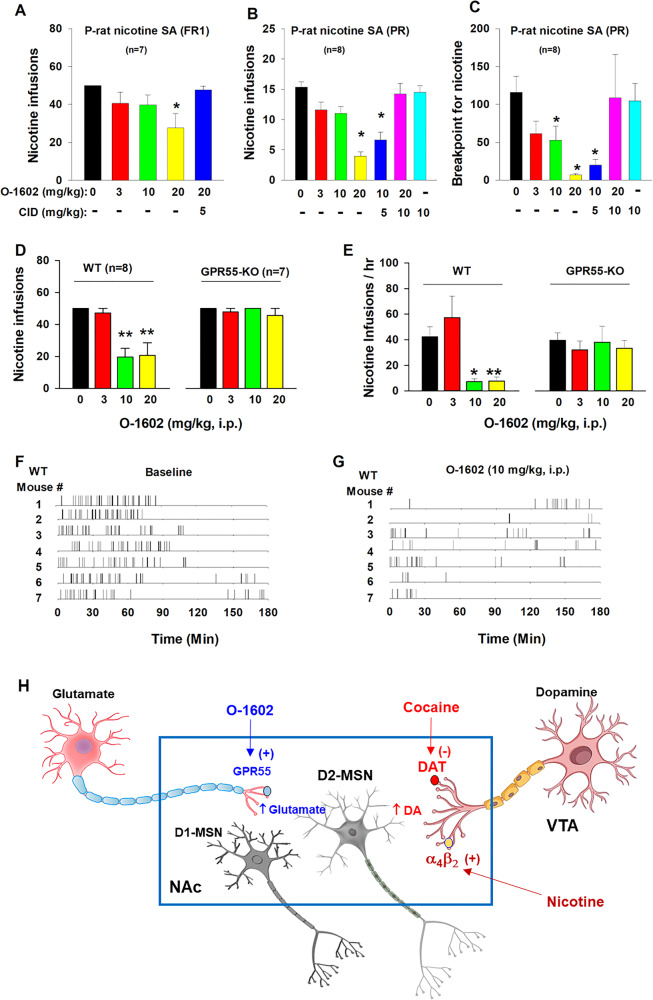
Effects of O-1602 on nicotine self-administration in rats and mice. **A** Systemic administration of O-1602 dose-dependently inhibited nicotine SA under FR1 reinforcement in P-rats. **B**, **C** O-1602 also inhibited nicotine SA under progressive-ratio (PR) reinforcement as assessed by the number of nicotine infusions (**B**) or breakpoint for nicotine SA (**C**). Pretreatment with CID 16020046 (CID), a selective GPR55 antagonist, blocked action produced by O-1602, while CID alone failed to alter nicotine SA under PR schedule. **D**, **E** Systemic administration of O-1602 (10 and 20 mg/kg) significantly inhibited nicotine SA in WT mice, but not in GPR55-KO mice. **F**, **G** Representative records of nicotine infusions after the vehicle (**F**) or O-1602 administration (**G**), illustrate that O-1602, at 10 mg/kg, caused cessation of nicotine self-administration. **H** A proposed working hypothesis explaining how GPR55 agonism reduces cocaine and nicotine self-administration. Briefly, cocaine or nicotine may increase extracellular DA levels in the NAc by blockade of DA transporter (DAT) or activation of α_4_β_2_ nicotinic receptors located on midbrain DA neurons or NAc DA terminals, respectively, which has been thought to underlie intravenous drug self-administration. Activation of GPR55 expressed on glutamate terminals projected from the cortex, and hippocampus increases glutamate release in the NAc, which subsequently counteracts DA effects on D2-MSNs where glutamate and DA produce opposite effects on neuronal activity. *p < 0.05, **p < 0.01, com*p*ared to the vehicle control group.

To further confirm the above findings in cocaine self-administration, we examined the effects of O-1602 on intravenous nicotine self-administration, as nicotine has been considered a weak reinforcer in self-administration when compared to cocaine [34]. We chose selectively bred alcohol-preferring rats (P-rats) in this experiment, as P-rats displayed significantly higher vulnerability than other strains of rats in obtaining nicotine self-administration in our previous report [34]. Here we found that O-1602 (3, 10, or 20 mg/kg) dose-dependently reduced nicotine self-administration under FR1 (Fig. 6A, *F*_4,23_ = 3.28, *p* < 0.05) or PR (Fig. 6B, *F*_5,42_ = 12.90, *p* < 0.001; Fig. 6C, *F*_5,42_ = 5.41, *p* < 0.001) reinforcement schedules. This inhibitory effect was blocked by co-administration of CID 16020046 (CID), a selective GPR55 antagonist. CID alone, at 10 mg/kg, failed to alter nicotine self-administration (Fig. 6B, C).

### O-1602 inhibits nicotine self-administration in WT, not GPR55-KO, mice

To verify whether the above action produced O-1602 is mediated by activation of GPR55, we used GPR55-KO mice as negative controls. Figure 6D, E shows that GPR55-KO mice did not differ in basal level of nicotine self-administration compared to WT mice (e.g., at 0 mg/kg). However, systemic administration of O-1602 (10, 20 mg/kg, i.p.) produced a robust reduction in nicotine self-administration in WT mice, as assessed by either the total number of nicotine infusions (Fig. 6D, *F*_3,21_ = 11.32, *p* < 0.001) or the rate of nicotine self-administration (Fig. 6E, *F*_3,21_ = 16.25, *p* < 0.001), but not in GPR55-KO mice (Fig. 6D, *F*_3,18_ = 0.71, *p* > 0.05; Fig. 6E, *F*_3,18_ = 1.06, *p* > 0.05). Figure 6F, G shows representative nicotine self-administration (infusion) records after vehicle or O-1602 treatment in WT mice, indicating that 10 mg/kg of O-1602 significantly reduced nicotine self-administration and altered the patterns of self-administration from a regular, evenly distributed pattern to an irregular, extinction-like pattern, suggesting that GPR55 agonism inhibits nicotine reward. Figure 6E shows a proposed working hypothesis through which O-1602 elevates extracellular glutamate that subsequently counteracts the action produced by cocaine- or nicotine-enhanced DA possibly in D2-expressing medium-spiny neurons (D2-MSNs).

### O-1602 has no effect on oral sucrose self-administration or open-field locomotion

To determine whether the O-1602-induced reduction in cocaine and nicotine self-administration was due to treatment-induced locomotor impairment, we observed the effects of O-1602 on open-field locomotion and non-drug (sucrose) self-administration. We found that systemic administration of the same doses of O-1602 neither altered sucrose self-administration as assessed by the total number of sucrose delivery (Fig. S7A, *F*_2,13_ = 1.05, *p* > 0.05) or the rate of sucrose deliveries (Fig. S7B, *F*_2,13_ = 1.38, *p* > 0.05) nor altered open-field locomotion (Fig. S7C, O-1602 treatment main effect, *F*_3,21_ = 0.53, *p* > 0.05; time main effect (F_11,77_ = 3.29, *p* < 0.001); treatment × time interaction, *F*_33,231_ = 0.31 *p* > 0.05). These findings suggest that O-1602 selectively inhibits cocaine and nicotine taking and seeking behavior.

## Discussion

In this study, we systematically examined the cellular distributions and functions of GPR55 in the brain. Our key findings are as follows: (1) GPR55 mRNA exhibits high expression in VgluT1^+^ or VgluT2^+^ glutamate neurons in the PFC, hippocampus, and thalamus, but not in midbrain DA neurons. (2) Two polyclonal GPR55 antibodies revealed GPR55-like immunostaining; however, the signal is not specific to GPR55. (3) Utilizing a fluorescent GPR55 ligand (T1117), we observed GPR55 binding in both VgluT1^+^ and VgluT2^+^ glutamate neurons in the PFC, hippocampus and red nucleus. (4) Systemic administration of the GPR55 agonist O-1602 failed to influence Δ^9^-THC-induced triad effects or DA-dependent brain-stimulation reward, but it dose-dependently inhibited cocaine-enhanced oICSS, cocaine self-administration under PR reinforcement, and nicotine self-administration under FR1 and PR reinforcement schedules. (5). In vivo microdialysis revealed that O-1602 dose-dependently increased extracellular glutamate, not DA, in the NAc. Lastly, (6) O-1602 neither affected open-field locomotion nor altered oral sucrose self-administration. Together, these findings suggest that brain GPR55 is mainly expressed in glutamate neurons and functionally modulates glutamate release and drug self-administration. Thus, GPR55 deserves further research as a novel therapeutic target for the treatment of cocaine or nicotine use disorders.

### Identification of GPR55 expression in glutamatergic neurons

The first important finding in this study is the identification of GPR55 mRNA in cortical and subcortical glutamate neurons, but not in midbrain DA neurons. This is consistent with a previous report indicating that GPR55 mRNA is colocalized with the neuronal marker NeuN, but not with an astrocytic marker GFAP or microglial marker Iba1 in the striatum [15]. Notably, a high density of GPR55 mRNA was detected in the cortex and hippocampus, while much lower GPR55 mRNA was detected in the thalamus, VTA, red nucleus, and striatum in the present study.

Unexpectedly, we found that the GPR55-immunostaining detected by two commonly used anti-GPR55 antibodies is not highly GPR55-specific, as it is still detectable in GPR55-KO mice or in WT mice in the presence of specific immune peptides. This contrasts with two previous reports indicating specific GPR55-immunostaining in the hippocampus and striatum in WT but not in GPR55-KO mice, using an anti-GPR55 antibody provided by Ken Mackie [17, 18]. The reasons for these conflicting findings are unclear. It may be related to the different epitopes of the antibodies. For example, the Abcam antibody we used in this study targets the C-terminal of human or bovine GPR55, while the epitopes of the Ken Mackie antibody [18] and the Cayman antibody we used in this study are undisclosed. In addition, it was recently reported that GPR55 is colocalized with substance P in striatal MSNs using another anti-GPR55 antibody (provided by Bioss). This antibody targets the transmembrane domains of human GPR55 [16]. However, the GPR55 specificity of this antibody is not tested in GPR55-KO mice. It is important to note that the low specificity of the GPR55 antibodies used does not necessarily imply that the signals detected are not related to GPR55. Rather, it suggests that, besides GPR55, the antibodies may also bind to other unintended targets. This is supported by our finding that the signal density detected by the Abcam GPR55 antibody is significantly lower in GPR55-KO mice than in WT mice.

Due to the concern of the antibody specificity, we then used a fluorescent CB1-GPR55 ligand (T1117) to examine GPR55 binding in CB1-KO mice. We had the same findings as those from RNAscope ISH. High-density GPR55 binding was detected in cortical VgluT1^+^ glutamate neurons and subcortical VgluT2^+^ glutamate neurons in the hippocampus, thalamus and red nucleus, but not in midbrain DA neurons. This finding is consistent with a previous report that the GPR55 is colocalized with VgluT1 in the hippocampus by double-label immunostaining [17]. Given that all three approaches (RNAscope ISH, IHC, and fluorescent T1117 binding) did not detect GPR55 signals in VTA DA neurons, we concluded that GPR55 may not directly regulate DA neuron activity and DA-related behavior. In contrast, all the above findings support GPR55 expression in glutamate neurons, suggesting that glutamatergic GPR55 may play a dominant role in GPR55 function under physiological and pharmacological conditions.

### GPR55 agonist failed to alter high dose Δ^9^-THC-induced triad effects

As previously reported, mice lacking CB1 or CB2 receptors exhibited a notable decrease, while GPR55-KO mice displayed a significant increase in classical cannabinoid effects, suggesting that GPR55 plays an opposing role to CB1 and CB2 receptors [22]. Consequently, we proposed that GPR55 agonists could be beneficial in treating cannabinoid use disorder by counteracting the effects mediated by CB1 and CB2 receptor activation. Surprisingly, pretreatment with O-1602 did not affect Δ^9^-THC-induced triad effects (analgesia, hypothermia, and catalepsy). The reasons for the lack of efficacy in this study remain unknown. This might be associated with the fact that both Δ^9^-THC and O-1602 are GPR55 agonists and exhibit similar high potency in GTPγS binding assays (Δ^9^-THC EC50: 8 nM; O-1602 EC50: 13 nM) [7, 16]. Thus, GPR55 agonism may not be capable of counteracting the effects produced by Δ^9^-THC but may have efficacy against actions produced by other drugs or cannabinoids lacking GPR55 binding profiles by themselves.

### GPR55 agonism inhibits cocaine and nicotine self-administration

To test the aforementioned hypothesis, we examined the impact of O-1602 on the actions of cocaine in oICSS and intravenous self-administration. Our findings revealed that GPR55 agonism significantly attenuated cocaine-enhanced brain stimulation reward and cocaine self-administration under PR reinforcement in rats, but not under FR1 or FR2 reinforcement schedules in both rats and mice. This observation may be attributed to the following factors: (1) the cocaine intake under FR1 and FR2 reinforcement schedules is much higher than that under PR reinforcement schedules; and (2) cocaine is a highly potent DA transporter inhibitor, producing a robust increase in extracellular DA. The robust increase in NAc extracellular DA caused by cocaine under FR1/FR2 reinforcement conditions could potentially compromise the action of glutamate produced by O-1602. Therefore, it is plausible that higher doses of O-1602 or more potent GPR55 agonists may be necessary to counteract cocaine action when cocaine is readily available under FR1 or FR2 reinforcement schedules.

Therefore, we proceeded to investigate the impact of O-1602 on the actions of nicotine, a substance considered relatively weaker in terms of reinforcing effects in drug self-administration [34]. Our findings revealed that O-1602 is notably more potent and effective in reducing nicotine self-administration under both FR1 and PR reinforcement. Importantly, this effect was blocked by GPR55 antagonism or the genetic deletion of GPR55, suggesting a GPR55-mediated effect. The observed reduction in nicotine self-administration is unlikely attributable to sedation or locomotor impairment, as O-1602, administered at equivalent doses, did not alter oral sucrose self-administration or open-field locomotion. This aligns with a previous report indicating that systemic administration of O-1602 failed to affect food intake and locomotion immediately (within 1–4 h) post-administration [35, 36]. It is noteworthy that an increase in food intake was observed 4–6 h after systemic or central administration [35], which has been shown to be unrelated to GPR55 but associated with the downregulation of the anorexigenic neuropeptide cocaine- and amphetamine-regulated transcript. The observation that O-1602 inhibits nicotine self-administration in both rats and mice is consistent with prior reports demonstrating its attenuation of CPP responses to morphine or nicotine, as well as naloxone-precipitated opioid withdrawal syndromes [24, 25].

We note that O-1602 is not a highly selective GPR55 agonist [9]. It is also a biased GPR18 agonist [37, 38], suggesting that non-GPR55 mechanisms may also contribute to the pharmacological action produced by O-1602 in this study. However, this possibility is unlikely as genetic deletion of GPR55 in GPR55-KO mice completely blocked the effects of O-1602 on nicotine self-administration, supporting critical involvement of GPR55 in the pharmacological action of O-1602.

### Glutamate mechanisms may underlie GPR55 modulation of drug self-administration

It is unknown exactly how GPR55 modulates cocaine or nicotine self-administration. Given that GPR55 agonism increases glutamate, but not DA, release in the NAc, we hypothesized that an enhanced glutamate transmission in the NAc may underlie the antagonism of O-1602 on drug self-administration (Fig. 6E). This is supported by several lines of evidence. First, activation of GPR55 increases intracellular Ca^++^ levels in neurons in the hippocampus, substantia nigra, and dorsal root ganglia [16, 17, 39], and facilitates glutamate release in the NAc (present study) and hippocampus [17]. Second, optical stimulation of glutamate terminals in the NAc projected from the paraventricular nucleus of the thalamus is not rewarding, but reward-attenuating or aversive [40]. Third, the elevation of extracellular glutamate levels in the NAc by blockade of glial glutamate transporters inhibits cocaine self-administration and cocaine intake, possibly via an action on postsynaptic D2 receptor-expressing medium-spiny neurons (D2-MSNs) [33].

It is well-documented that drug self-administration is primarily maintained through a DA-dependent mechanism [41, 42]. Cocaine increases extracellular DA levels in the NAc by blockade of DA transporter (DAT), while nicotine increases NAc DA release mainly by activation of α_4_β_2_ nicotinic receptors located on midbrain DA neurons [41, 43]. Subsequently, DA activates D1 receptor-expressing medium-spiny neurons (D1-MSNs) via excitatory Gs-coupled proteins, while it inhibits D2-MSNs via inhibitory Gi-coupled proteins. Both the actions in D1-MSNs and D2-MSNs contribute to the rewarding effects [44]. O-1602, by increasing NAc glutamate release, may functionally counteract DA action in D2-MSNs [33]. We therefore propose that this glutamate mechanism may partially explain how GPR55 agonism produces inhibitory effects on cocaine or nicotine self-administration (Fig. 6E). Certainly, other possibilities cannot be excluded.

We note another study reporting that the microinjection of palmitoylethanolamide (PEA), an endogenous agonist for both GPR55 and PPARα [45, 46], into the ventral hippocampus (vHipp), increased the firing and bursting activity of VTA DA neurons. This effect was found to be blocked by a selective GPR55 antagonist, CID 16020046 [47], suggesting a potential involvement of a DA-dependent mechanism. However, in our current study, we did not observe GPR55 expression in VTA DA neurons. Moreover, we did not detect significant modulation of extracellular DA or DA-dependent oICSS behavior by O-1602, indicating that an indirect mechanism may be involved in that report. This interpretation is supported by the findings from the mentioned study, wherein it was reported that an NMDA receptor antagonist could block the increase in VTA DA neuron firing induced by intra-vHipp PEA. Additionally, locally enhanced DA levels by PEA failed to alter morphine-induced CPP response [47].

In conclusion, this study systematically investigated the cellular distributions of GPR55 in the mouse brain and its functional implications in cannabinoid actions and substance use disorders. Our findings indicate that GPR55 is predominantly expressed in cortical and subcortical glutamate neurons, with minimal expression in midbrain DA neurons. The activation of GPR55 by O-1602 increased glutamate release in the NAc and, in a dose-dependent manner, inhibited cocaine or nicotine self-administration under FR and/or PR reinforcement conditions in both rats and mice. These results suggest that a glutamate-dependent mechanism may underlie the pharmacological effects of GPR55 agonism in drug self-administration. Consequently, GPR55 warrants further investigation as a therapeutic target for the treatment of cocaine and nicotine use disorders.

### Supplementary information

GPR55 - SI

Suppl. Fig. 1 -GPR55-DAT-RNAscope

Suppl. Fig. 2 - GPR55 - RNAscope - High magnification

Suppl. Fig. 3 - RNAscope - Hipp +

Suppl. Fig. 4 - THC-Triad behavior

Suppl. Fig. 5 - GPR55-IHC-Abcam Ab

Suppl. Fig. 6 - GPR55-IHC-Cayman Ab

Suppl. Fig. 7 - Sucrose SA + Locomotion

## Data Availability

The raw data in this manuscript is available upon request
