# Supplementary material for: GPR55 is expressed in glutamate neurons and functionally modulates drug taking and seeking in rats and mice
Source: Transl Psychiatry. 2024 Feb 19;14:101. doi: 10.1038/s41398-024-02820-3 (PMC10876975; doi:10.1038/s41398-024-02820-3)
Supplement: Supplementary file 1 — GPR55 - SI [file 41398_2024_2820_MOESM1_ESM.docx]

Translational Psychiatry

**Supplementary Information**

**GPR55 is expressed in glutamate neurons and functionally modulates drug taking and seeking in rats and mice**

Yi He^1*^, Hui Shen^2*^, Guo-Hua Bi^1,3^, Hai-Ying Zhang^1^, Omar Soler-Cedeño^1,4^, Hannah Alton^1,3^, Yihong Yang^2^, Zheng-Xiong Xi^1#^

**Materials and Methods**

**Animal**

Male adult Long-Evans (LE) and alcohol-preferring rats (P-rats, obtained from Indiana University Medical Center) were used in the present study. Male and female GPR55-KO mice were purchased from Lexicon Pharmaceuticals (Cat. #: Lexko-0261 , B6;129S-Gpr55^tm1Lex^/Mmnc, The Woodlands, Texas, USA). Heterozygous CB_1_^+/-^ breeding pairs were kindly provided by Dr. Andreas Zimmer during his tenure at the National Institute of Mental Health (Bethesda, MD) (1). DAT-cre mice (Slc6a3tm1(cre)Xz/J) were obtained from the Jackson Laboratory (Stock No: 020080).  All of the transgenic mice and their wild-type littermates were bred in the animal facility of the National Institute on Drug Abuse (NIDA) Intramural Research Program (IRP). All of the animals were held on a reversed 12:12 light/dark cycle (lights on at 7:00 p.m.). The animal care and the experimental procedure were conducted in accordance with the Guide for the Care and Use of Laboratory Animals and were approved by the Animal Care and Use Committee of NIDA.

**Drugs**

Cocaine and nicotine hydrochloride were obtained through the NIDA Pharmacy. The dose of nicotine hydrochloride salt was converted to freebase nicotine (MW=162.2 g/mol) by dividing the nicotine hydrochloride (162.2/198.69) when preparing nicotine solution for intravenous self-administration. O-1602 was supplied by Cayman Chemical (Cat#: 10006803) as a solution in methyl acetate. Before intraperitoneal (i.p.) administration, O-1602 methyl acetate was evaporated under a gentle stream of nitrogen, and then O-1602 was resuspended in saline solution, which contains 5% cremophor EL (CrEL) (Sigma-Aldrich, # C5135). The selective GPR55 antagonist CID 16020046 (CID, Cat#: 4959) and Tocrifluor T1117 (a fluorescent cannabinoid ligand, Cat. # 2540) were purchased from Tocris Bioscience.

**Experiment 1: GPR55 RNAscope ISH**

Given the important role of dopamine and glutamate in cannabinoid action and drug addiction (2, 3), we first examined GPR55 gene expression in midbrain DA neurons and cortical/subcortical glutamate neurons using RNAscope ISH assays to examine the co-localization of GPR55 mRNA and tyrosine hydroxylase (TH) mRNA, dopamine transporter (DAT) mRNA, or vesicular glutamate transporter 1 (VgluT1) mRNA. Mice were deeply anesthetized, and the whole brain was removed and rapidly frozen on dry ice. Fresh-frozen tissue sections (14 μm thick) were mounted on positively charged microscopic glass slides (Fisher Scientific) and stored at −80°C until RNAscope ISH assays could be performed. Multiple target gene-specific RNAscope probes were designed and provided by Advanced Cell Diagnostics (Newark, CA, USA). The riboprobes were used to observe the cellular distributions of GPR55 mRNA in VgluT1-expressing glutamate neurons and TH-/DAT-expressing DA neurons, which include the GPR55 RNAscope probe (Cat #: 318231, targeting 2-907 bp of the mouse GPR55 mRNA sequence, NM _001033290.2), the VgluT1 RNAscope probe (Cat #: 503518-C3, targeting 621–1021 bp of the *Mus musculus* VgluT1 mRNA sequence, NM_182993.2), the TH-specific RNAscope probe (Cat #: 317621-C2, targeting 483–1,603 bp of the *Mus musculus* TH mRNA sequence, NM_009377.1), and the DAT RNAscope probe (Mm-Slc6a3-C2; cat. no. 315441; targeting 1,486–2,525 bp of the *M. musculus* solute carrier family 6 DAT mRNA sequence). The RNAscope mRNA-staining steps were performed following the manufacturer's protocols. Stained slides were cover-slipped with fluorescent mounting medium (ProLong Gold Anti-fade Reagent P36930; Life Technologies) and scanned into digital images with an Olympus FluoView FV1000 confocal microscope at 40× or 60× magnification using manufacturer-provided software.

**Experiment 2: GPR55 immunohistochemistry (IHC) assays**

We then used IHC to detect GPR55 receptor expression in VTA DA neurons in WT and GPR55-KO mice. The IHC procedures were performed as reported previously (4).  Briefly, mice were deeply anesthetized with 100 mg/kg pentobarbital and transcardially perfused with cold saline, followed by 4% paraformaldehyde in 0.1-M phosphate buffer. Brain tissue was transferred to 20% sucrose in phosphate buffer at 4°C overnight. Coronal sections were cut at 25 μm on a cryostat (CM3050S, Leica Microsystems Nussloch GmbH, Nussloch, Germany). Tissue sections containing the VTA were blocked and floated in 5% bovine serum albumin and 0.5% Triton X-100 phosphate buffer for 2 h at room temperature. Dual-labeling IHC was performed using a GPR55 polyclonal antibody (Caymen, #10224, 1:250; Abcam, #ab174700 with the epitope on C-terminal aa#245~319, 1:250) and an anti-tyrosine hydroxylase (anti-TH) monoclonal antibody (1:500; Millipore, Billerica, MA, USA) in the presence or absence of the GPR55 blocking peptide (Caymen, Cat.# 10225; or Abcam, ab50540, 1: 1 (v/v) of the antibody: the peptide ratio). Sections were washed and incubated with a mixture of secondary antibodies, goat anti-rabbit Alexa 488 for GPR55, and goat anti-mouse Alexa 568 for TH (Millipore, #MAB318; 1:500) in 5% bovine serum albumin and 0.5% Triton X-100 phosphate buffer for 2 h at room temperature. Sections were washed, mounted, and coverslipped. Fluorescent images were captured using a fluorescence microscope (Nikon Eclipse 80i) equipped with a digital camera (Nikon Instruments Inc., Melville, NY, USA). All images were captured under identical optical conditions.

**Experiment 3: Fluorescent GPR55-ligand binding assays**

Given that the GPR55 antibody displayed poor receptor specificity (see the results section below), we next used a novel fluorescent GPR55 ligand (Tocrifluor T1117, i.e., T1117), a GPR55 agonist (5, 6), to detect GPR55 expression in VTA DA neurons. Given that Tocrifluor T1117 is a fluorescent analog of AM251 (a selective CB1 receptor antagonist) and has been shown to have low binding affinity to CB1 receptor (5, 6), we used CB1-KO mice to exclude its possible binding to CB1 receptor, and therefore, makes it specifically binding to GPR55. The procedures for T1117 binding were the same as the GPR55-IHC described above except T1117 (500 nM) replaced the GPR55 antibody used in the above IHC. To examine T1117 binding on cortical and subcortical glutamate neurons, both the VgluT1 (guinea pig, 1:2000, Synaptic Systems, RRID: [AB_887878](https://scicrunch.org/resolver/AB_887878)) and VgluT2 (rabbit, 1:1000, Synaptic Systems, RRID: [AB_887883](https://scicrunch.org/resolver/AB_887883)) antibodies were used in the IHC assays. To determine its possible binding on VTA DA neurons, we used an anti-TH monoclonal antibody (1:500; Millipore, Billerica, MA, USA) to label midbrain DA neurons.

**Experiment 3: Δ^9^-THC-induced triad effects**

To determine whether the GPR55 agonist O-1602 is able to alter Δ^9^-THC-induced triad effects, we measured the nociceptive response to heat stimulus (analgesia), body temperature, hypothermia, cataleptic behavior and rotarod locomotor performance after systemic administration of 30 mg/kg Δ^9^-THC in the absence (vehicle) or presentce of 10 mg/kg or 20 mg/kg O-1602 in WT mice (n=8). A within-subjects design was used in this experiment. Each mouse received three drug injections with different drug doses in a counter-balanced order. The effects of O-1602 on Δ^9^-THC-induced triad effects were evaluated.

***Analgesia assessment:*** Analgesia (defined as a decrease in thermal pain sensitivity) was assessed using a hot-plate device (Model 39, IITC Life Science Inc., CA, USA). A mouse was placed inside a transparent cage on the hot plate (52 ± 0.2°C). The latency to the first sign of thermal nociception (licking, stomping the hind paw or jumping) was measured in seconds. The cut-off time for the test was 60 s to avoid tissue damage.

**Hypothermia assessment:** Hypothermia (defined as a decrease in rectal temperature; °C) was measured with a thermometer connected to a lubricated RET-2 rectal probe (Harvard Apparatus, Holliston, MA, USA) that was inserted into the rectum to a depth of 2 cm.

**Catalepsy assessment:** Catalepsy, defined as an impaired capacity to initiate movements, was measured using an elevated bar test. Mice were hung by their front paws from a rubber coated metal bar (12-cm length) that was fixed horizontally at a height allowing their hind paws to just touch the floor. The latency to descend from the bar (i.e. when the two forepaws touched the floor) or when 3 min elapsed (i.e. cut-off time) was measured.

**Experiment 4: Optogenetic intracranial self-stimulation (oICSS)**

To determine whether O-1602 itself is rewarding and is able to alter DA-dependent behavior, we observed the effects of O-1602 on oICSS, a new animal model used to evaluate drug modulation of DA-dependent behavior.

*Surgery*: DAT-cre mice were anesthetized by an i.p. injection of ketamine/xylazine and then microinjected with adeno-associated virus (AAV) carrying ChR2-GFP (AAV5-EF1α-DIO-hChR2-GFP), followed by implantations of custom-made optical fibres (200-μm inner diameter, N.A. 0.22; Doric Lenses, Quebec, Canada) targeted at the VTA (AP − 3.2, ML ± 0.1, DV – 3.7) (7, 8).

*The procedures for oICSS*: The oICSS procedures were the same as reported previously (7). DAT-cre mice (*n* = 6), with ChR2 expression targeting the DA neurons in the ventral tegmental area (VTA), were connected to a 473-nm wavelength laser (OEM Laser Systems, UT) by two sheathed optic fibres (200-μm core diameter, Precision Fiber Products, Chula Vista, CA, USA) and FC/FC fibre rotary joint (Doric Lenses Inc., Quebec, Canada). The total output of the laser was adjusted to ∼10 mW transmittance into the brain. During each 1-hr session, each press on the active lever activated the light above the lever and delivered a 1-s pulse train of blue light (473 nm, 10 mW, 5 ms pulse duration, and 50 Hz) depolarizing VTA DA neurons. Pressing the inactive lever yielded no stimulation. Once animals' responding stabilized (<20% variability in responding for at least three consecutive sessions), a multiple stimulation frequency schedule was introduced. Every 10 min, stimulation frequency was decreased from 100 to 50, 25, 10, 5, and finally to 1 Hz, and lever presses at each frequency were counted. The testing phase began once stable oICSS responding was achieved with <20% variation across three consecutive sessions. Mice received an i.p. injection of vehicle or one dose of O-1602 (10 or 20 mg/kg) 15 min prior to the test session and allowed to lever press for oICSS. After each test, mice received additional oICSS sessions until a new baseline was established and later were re-tested with a different dose of the drug. In addition, we also evaluated the effects of O-1602 (0, 10, 20 mg/kg, i.p., 30 min prior to 10 mg/kg cocaine) on cocaine action in oICSS in a separate groups of mice. After completion of the above behavioural experiment, immunohistochemistry assay was used to verify AAV-ChR2-EGFP expression in VTA DA neurons in DAT-cre mice using the methods we reported previously (9).

**Experiment 5: *In vivo* microdialysis in P-rats**

To determine whether a DA- or glutamate-dependent mechanism underlies O-1602 action, we used *in vivo* microdialysis to measure DA/glutamate response to O-1602 in P-rats. We chose P-rats in this experiment because P-rats displayed high vularability to nicotine in self-administration (10) and O-1602 displayed high efficacy in reducing nicotine self-administration in this strain of rats (see the main text). The purpose of this experiment was to determine whether a DA- or glutamate-dependent mechanism may underlie the antagonism of O-1602 on nicotine self-administration. The procedures for intracranial guide cannula implantation for microdialysis were as reported previously (11). Guide cannulae (20 gauge; Plastics One, Roanoke,VA) were surgically implanted into the NAc (anteroposterior, +1.6 mm; mediolateral, ±2.0 mm; dorsoventral, -4.0 mm; angled 6° from vertical) using standard surgical and stereotaxic techniques. Microdialysis probes were inserted into the NAc 12 h before the experiment to minimize damage-induced neurotransmitter release.

To collect the extracellular fluid sample, the animals were lightly anesthetized with isoflurane and the microdialysis probes (SciPro Inc., MAB 6.14) were gently inserted into the guide cannulas in the NAc. To minimize damage-induced neurotransmitter release, implanted probes were perfused overnight with artificial cerebrospinal fluid (aCSF) before the microdialysis experiment began. On the microdialysis day, sample collection started with 1 hour of baseline by perfusing aCSF at the flow rate of 2.0 μl/min. Then O-1602 was systematically administered by intraperitoneal injection (3 and 10 mg/kg). Dialysis samples were collected every 10 min into an Eppendorf tube containing 10 μL of 0.5 M perchloric acid, which is for preventing neurotransmitter degradation. All samples were frozen at -80°C until analyzed by HPLC. DA and glutamate in the dialysis samples were measured, respectively, with an electrochemical detection system (ESA, Chelmsford, MA) and a fluorescence amino acid detection system according to the protocols we reported previously (11).

**Experiment 6: Drug self-administration in rats**

*Surgery* The procedures for jugular catheter surgery and nicotine self-administration were as previously reported (10). Briefly, rats were anesthetized by an i.p. injection of ketamine/xylazine, and catheters, constructed of microrenathane (Braintree Scientific Inc., Braintree, MA, USA), were inserted into the right jugular vein. After being sutured into place, the catheter was passed s.c. to the top of the skull and exited and attached to a connector (a modified 24-g cannula; Plastics One, Roanoke, VA, USA). The connector was then mounted onto the skull using jewelry stainless-steel screws and dental acrylic. To prevent clogging, the catheters were flushed daily with a gentamicin-heparin-saline solution (30 IU/ml heparin) (ICN Biochemicals, Cleveland, OH, United States).

*Self-administration procedures*: The procedure for drug self-administration was the same as we reported previously (10, 12). After recovery from surgery, rats were placed in the self-administration chambers (Model MED-008-CT-B1, Med Associates) and trained to self-administer cocaine (0.5 or 1.0 mg/kg/infusion) or nicotine (30 µg/kg/infusion) daily under fix ratio (FR1, FR2) and progressive-ratio (PR) schedules during the dark phase. Each suppression on the lever paired with drug injection results in single intravenous delivery of a volume of 80 µl cocaine or nicotine solution over 5 sec along with a combined light-tone cue above the lever. Each training session lasts 3 hours or ends immediately when maximally 50 infusions have been taken to avoid cocaine overdose. After self-administration was stabilized, defined as ≥10 nicotine infusions per 3 h session; <20% variability in daily nicotine infusions across two consecutive sessions; an active/inactive lever pressing ratio exceeding 2:1, the effects of O-1602 (in the presence or absence of CID 16020046) on cocaine or nicotine self-administration were evaluated under an FR1 or FR2 reinforcement schedules. After each test, the animals continued daily drug self-administration until a stable baseline was re-established. The order of drug doses was counterbalanced. The time intervals between different drug/dose tests were 3~5 days.

*PR self-administration*: After the completion of the drug tests under FR1 schedule, the animals with stable drug self-administration were combined with an additional group of naïve rats and continued for cocaine or nicotine self-administration under a progressive ratio (PR) reinforcement schedule according to the protocols we reported previously (13). During the PR schedule, the delivery of each successive reward was accompanied by an increasing number of lever presses in ascending order: 1, 2, 4, 6, 9, 12, 15, 20, 25, 32, 40, 50, 62, 77, 95, 118, 145, 178, 219, 268, 328, 402, 492 and 603 until the break-point was reached. The break-point was defined as the maximal workload (i.e., number of lever presses) completed for the last drug infusion prior to a 1-h period during which no infusions were obtained by the animal. Animals self-administered cocaine or nicotine daily under the PR reinforcement conditions until day-to-day variability in break-points fell within 1-2 ratio increments for three consecutive days. After a stable break-point was established, subjects were assigned randomly to different subgroups to determine the effects of O-1602 (3, 10, 20 mg/kg, i.p.) or vehicle (15 minutes prior to test) on PR break-point for drug self-administration. To determine the receptor mechanism by which O-1602 produces its effects on nicotine self-administration, CID, a selective GPR55 antagonist (3, 10 mg/kg) was administrated 15 min prior to the injection of O-1602. Since it is relatively difficult to re-establish a stable break-point level after each drug test, we used a between-subjects design to determine the dose–response effects of O-1602 on break-point for cocaine or nicotine self-administration.

**Experiment 7: Intravenous drug self-administration in mice**

To determine whether the pharmacological action of O-1602 is mediated selectively by activation of GPR55, we further observed the effects of O-1602 on cocaine or nicotine self-administration in wildtype (WT) and GPR55-KO mice.

Prior to and during drug self-administration, GPR55 WT and KO mice were mildly food restricted to maintain their body around 85% of their free-feeding values to increase their operant conditioning responses. Before being given access to nicotine self-administration, the mice were trained to orally self-administer 5% sucrose (FR1) for three days (1 hr/daily session) to facilitate operant responses for reward seeking. Then animals were subjected to catheterization in their right jugular vein and allowed recovery for 5~7 days. Drug self-administration was conducted in mouse operant chambers (Model ENV-307A, Med Associates) at the dose of 30 µg/kg/infusion of nicotine (free base) or 0.5 mg/kg/infusion of cocaine. Pressing on the active lever will trigger the infusion pump to deliver 15 µl of nicotine or cocaine solution over 4.2 s and simultaneous presentation of the cue light and tones. The procedures of mouse self-administration training under FR1 reinforcement schedule and the criteria of stable self-administration were identical to those described above in rats except the minimal number of nicotine infusions ≥20 infusions per session in mice. The effects of O-1602 on mouse self-administration were evaluated in the same way as described above in rats under the same experimental conditions.

**Experiment 8: Oral sucrose self-administration in mice**

To determine whether the pharmacological action of O-1602 is drug-specific, we also observed the effect of O-1602 on non-drug (sucrose) self-administration in WT and GPR55-KO mice. The procedures for oral sucrose self-administration were identical to those described above for mouse i.v. drug self-administration except that there was no i.v. catheterization surgery and each active lever pressing led to a delivery of 5% sucrose solution (20 µL per delivery) into a liquid food receptacle installed on the wall of the chamber. Daily FR1 schedule was employed throughout the whole training procedure. A maximum of 100 sucrose deliveries was allowed during each 3-hour training session. After the behavior was stabilized, the animals randomly received one of three doses of O-1602 (vehicle, 10, 20 mg/kg, i.p.) 15 min prior to the self-administration test. A within-subjects design was used in this experiment. After each drug test, the animals continued daily sucrose self-administration until a stable baseline was re-established. The order of drug doses was counterbalanced. The time intervals between different drug/dose tests were 3~5 days.

**Experiment 9: Locomotor activity in mice**

Lastly, to determine whether the reduction in drug self-administration was due to locomotor impairment after O-1602 administration, we examined the effects of O-1602 on open-field locomotion in mice. Before the drug test, rats (*n* = 8) were placed in open-field locomotor chambers (Accuscan, Columbus, OH, USA) for habituation for 2–3 days (1 hr per day). On the following test days, mice were randomly treated with one of the O-1602 doses (0, 10, or 20 mg/kg, i.p.) after 1-hr pre-drug habituation. The time intervals between tests were 2~3 days. Following each injection, locomotor activity was recorded for additional 2 hrs in 10-min bins, and the traveled distance was used to evaluate the effects of O-1602 on locomotor activity.

**Data analysis**

Data analyses and graphing were accomplished using SigmaPlot software (version 13.0, Systat Software Inc., CA, USA). One-way repeated measures (RM) ANOVAs were used for comparing the effects of treatment on self-administration. Two-way RM ANOVAs were performed for analyzing the effect of treatments on locomotion, extracellular DA/glutamate, and oICSS behavior. Graphs were made based on the results reported as mean ± SEM. *p*<0.05 was defined as a statistically significant difference.

Animal group sizes were chosen based on a power analysis (*n* ≥ 8 per group) and extensive previous experience with the animal models used. The group size is the number of independent values (individual animals), and statistical analysis was done using these independent values. No data points were excluded from the analysis in any experiment. The investigators were blinded to the group allocation during the experiments and when assessing the outcome. To validate the use of parametric statistics, we performed a Shapiro Wilk Test for data normality evaluation and Levene’s test for homogeneity for between-subject ANOVA. Estimation statistics were used when necessary (when data were not normally distributed ([www.estimationstats.com](http://www.estimationstats.com/))).

**References:**

1. A. Zimmer, A. M. Zimmer, A. G. Hohmann, M. Herkenham, T. I. Bonner, Increased mortality, hypoactivity, and hypoalgesia in cannabinoid CB1 receptor knockout mice. *Proc Natl Acad Sci U S A* **96**, 5780-5785 (1999).

2. E. Galaj, A. H. Newman, Z. X. Xi, Dopamine D3 receptor-based medication development for the treatment of opioid use disorder: Rationale, progress, and challenges. *Neurosci Biobehav Rev* **114**, 38-52 (2020).

3. C. J. Jordan, J. Cao, A. H. Newman, Z. X. Xi, Progress in agonist therapy for substance use disorders: Lessons learned from methadone and buprenorphine. *Neuropharmacology* **158**, 107609 (2019).

4. H. Y. Zhang *et al.*, Cannabinoid CB2 receptors modulate midbrain dopamine neuronal activity and dopamine-related behavior in mice. *Proc Natl Acad Sci U S A* **111**, E5007-5015 (2014).

5. C. J. Daly *et al.*, Fluorescent ligand binding reveals heterogeneous distribution of adrenoceptors and 'cannabinoid-like' receptors in small arteries. *Br J Pharmacol* **159**, 787-796 (2010).

6. R. K. Paul *et al.*, (R,R')-4'-methoxy-1-naphthylfenoterol targets GPR55-mediated ligand internalization and impairs cancer cell motility. *Biochem Pharmacol* **87**, 547-561 (2014).

7. B. A. Humburg *et al.*, Optogenetic brain-stimulation reward: A new procedure to re-evaluate the rewarding versus aversive effects of cannabinoids in dopamine transporter-Cre mice. *Addict Biol* **26**, e13005 (2021).

8. E. Galaj *et al.*, Beta-caryophyllene inhibits cocaine addiction-related behavior by activation of PPARalpha and PPARgamma: repurposing a FDA-approved food additive for cocaine use disorder. *Neuropsychopharmacology* **46**, 860-870 (2021).

9. X. Han *et al.*, Cannabinoid CB1 Receptors Are Expressed in a Subset of Dopamine Neurons and Underlie Cannabinoid-Induced Aversion, Hypoactivity, and Anxiolytic Effects in Mice. *J Neurosci* 10.1523/JNEUROSCI.1493-22.2022 (2022).

10. X. F. Wang *et al.*, R-modafinil attenuates nicotine-taking and nicotine-seeking behavior in alcohol-preferring rats. *Neuropsychopharmacology* **40**, 1762-1771 (2015).

11. Z. X. Xi *et al.*, Cannabinoid CB1 receptor antagonist AM251 inhibits cocaine-primed relapse in rats: role of glutamate in the nucleus accumbens. *J Neurosci* **26**, 8531-8536 (2006).

12. Y. He *et al.*, beta-Caryophyllene, a dietary terpenoid, inhibits nicotine taking and nicotine seeking in rodents. *Br J Pharmacol* **177**, 2058-2072 (2020).

13. Z. X. Xi *et al.*, Cannabinoid CB1 receptor antagonists attenuate cocaine's rewarding effects: experiments with self-administration and brain-stimulation reward in rats. *Neuropsychopharmacology* **33**, 1735-1745 (2008).

**Figure legends**

**Figure S1** (related to Fig. 1): Representative RNAscope ISH images in WT and GPR55-KO mice. GPR55 mRNA signal was detected in WT, not in GPR55-KO, mice. However, GPR55 is not colocalized with dopamine transporter (DAT), another DA neuronal marker, in VTA DA neurons in WT mice.

**Figure S2** (related to Fig. 1C): Representative RNAscope ISH image under high magnification (60×), illustrating colocalization of GPR55 mRNA (red) and VgluT1 mRNA (green) in prefrontal cortex glutamate neurons. Blue color shows nucleus DAPI staining.

**Figure S3** (related to Fig. 1): Representative GPR55 RNAscope ISH images, illustrating GPR55 mRNA expression and its co-localization with VgluT1 in subcocical regions including the hippocampus (Hippo) (**A**), thalamus (**B**) and striatum (**C**).

**Figure S4** (related to Fig. 2): GPR55 IHC assays in WT and GPR55-KO mice. Using the GPR55 antibody purchased from Abcam, we detected GPR55-like immunostaining in the VTA of WT (**A**) and GPR55-KO (**B**) mice. Co-administration of the immune peptide failed to block the immunostaining (**C**), suggesting that this antibody is not highly GPR55-specific.

**Figure S5** (related to Fig. 2): GPR55 IHC assays with a GPR55 antibody purchased from Cayman Chemicals detected GPR55-like immunostaining in the VTA of WT mice in the absence (**A**) or presence of the immune peptide (**B**). Notably, GPR55-like staining is not colocalized with TH-immunostaining under any experimental conditions. The immune peptide used to generate the antibody failed to block the immunostaining, suggesting the signal detected is not GPR55-specific.

**Figure S6**: Effects of O-1602 on Δ^9^-THC-induced triad effects in mice. Systemic administration of Δ^9^-THC produced significant analgesia (as assessed by hot-plate test) (**A**), hypothermia (**B**) and catalepsy (**C**), which was not altered significantly by pretreatment with O-1602 (10, 20 mg/kg, i.p., 15 min prior to Δ^9^-THC injection).

**Figure S7** (related to Figs. 5-6): Effects of O-1602 on oral sucrose self-administration and open-field locomotion in mice. O-1602, at the same doses that inhibits nicotine self-administration, neither altered oral sucrose self-administration as assessed by the total number of sucrose deliveries (**A**) or the rate of sucrose self-administration (**B**) nor altered open-field locomotion (**C**).
