# Supplementary figures and images for: GPR55 is expressed in glutamate neurons and functionally modulates drug taking and seeking in rats and mice
Source: Transl Psychiatry. 2024 Feb 19;14:101. doi: 10.1038/s41398-024-02820-3 (PMC10876975; doi:10.1038/s41398-024-02820-3)

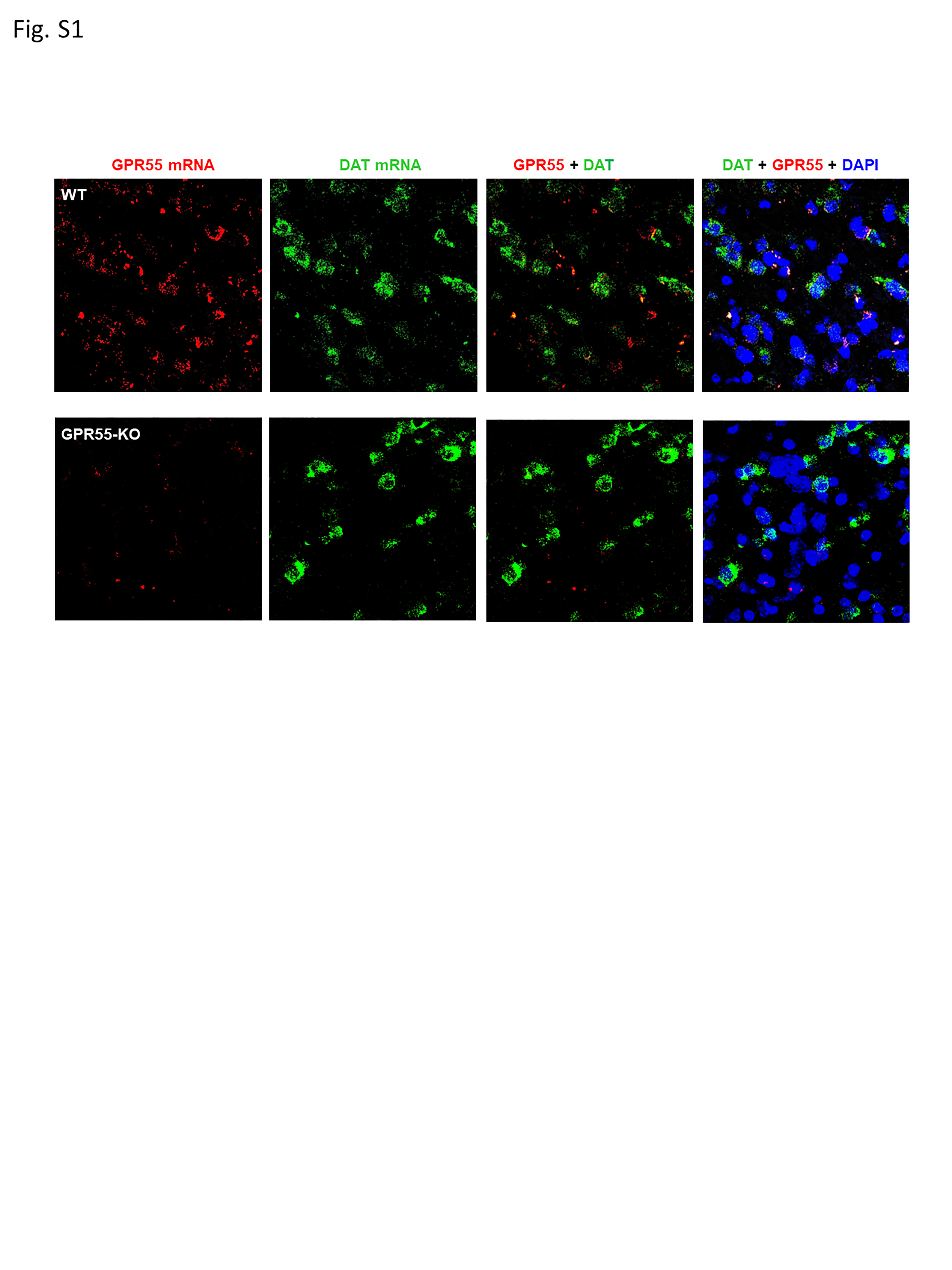

Supplement: Supplementary file 2 — Suppl. Fig. 1 -GPR55-DAT-RNAscope [file 41398_2024_2820_MOESM2_ESM.tif]

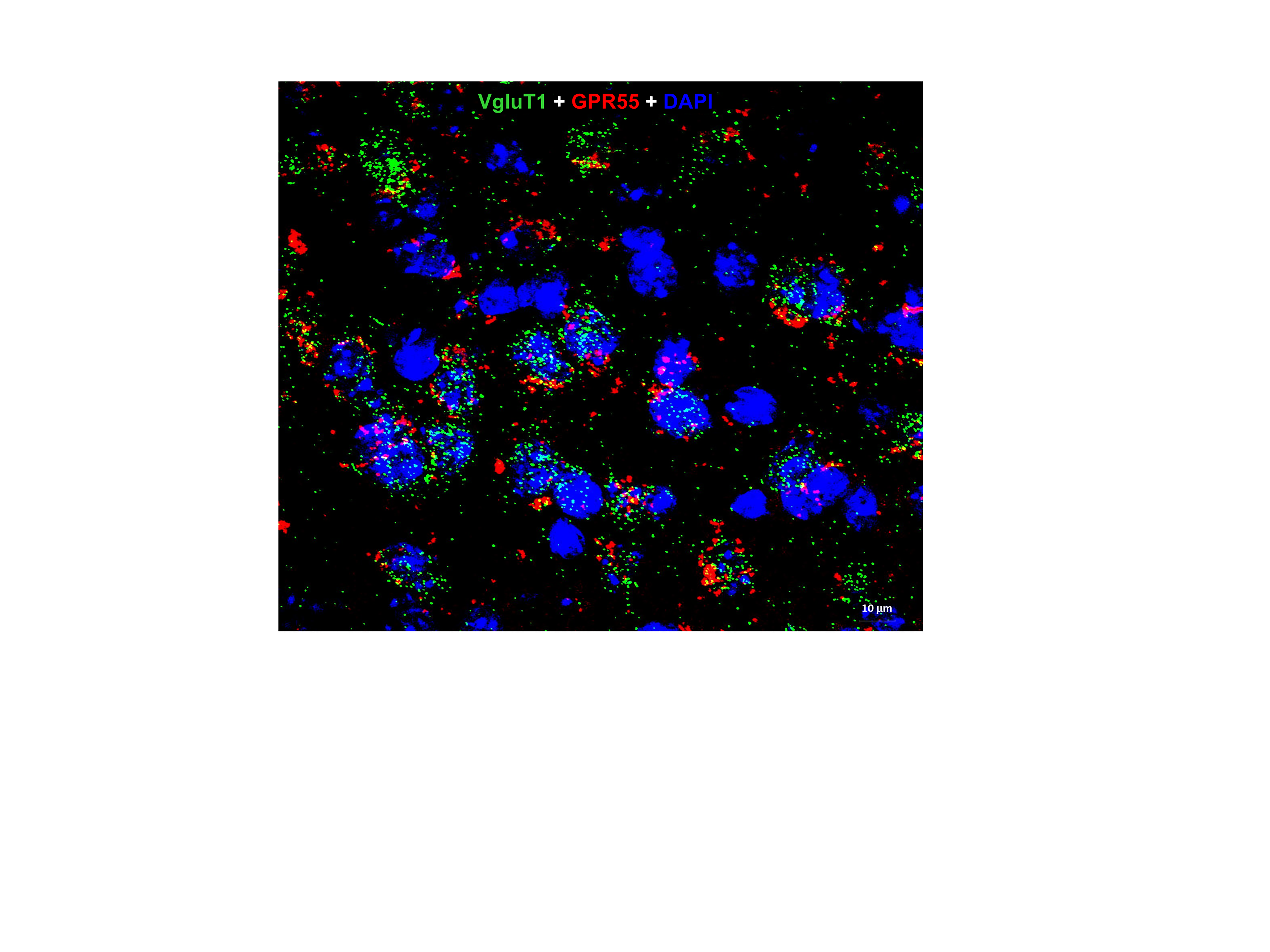

Supplement: Supplementary file 3 — Suppl. Fig. 2 - GPR55 - RNAscope - High magnification [file 41398_2024_2820_MOESM3_ESM.tif]

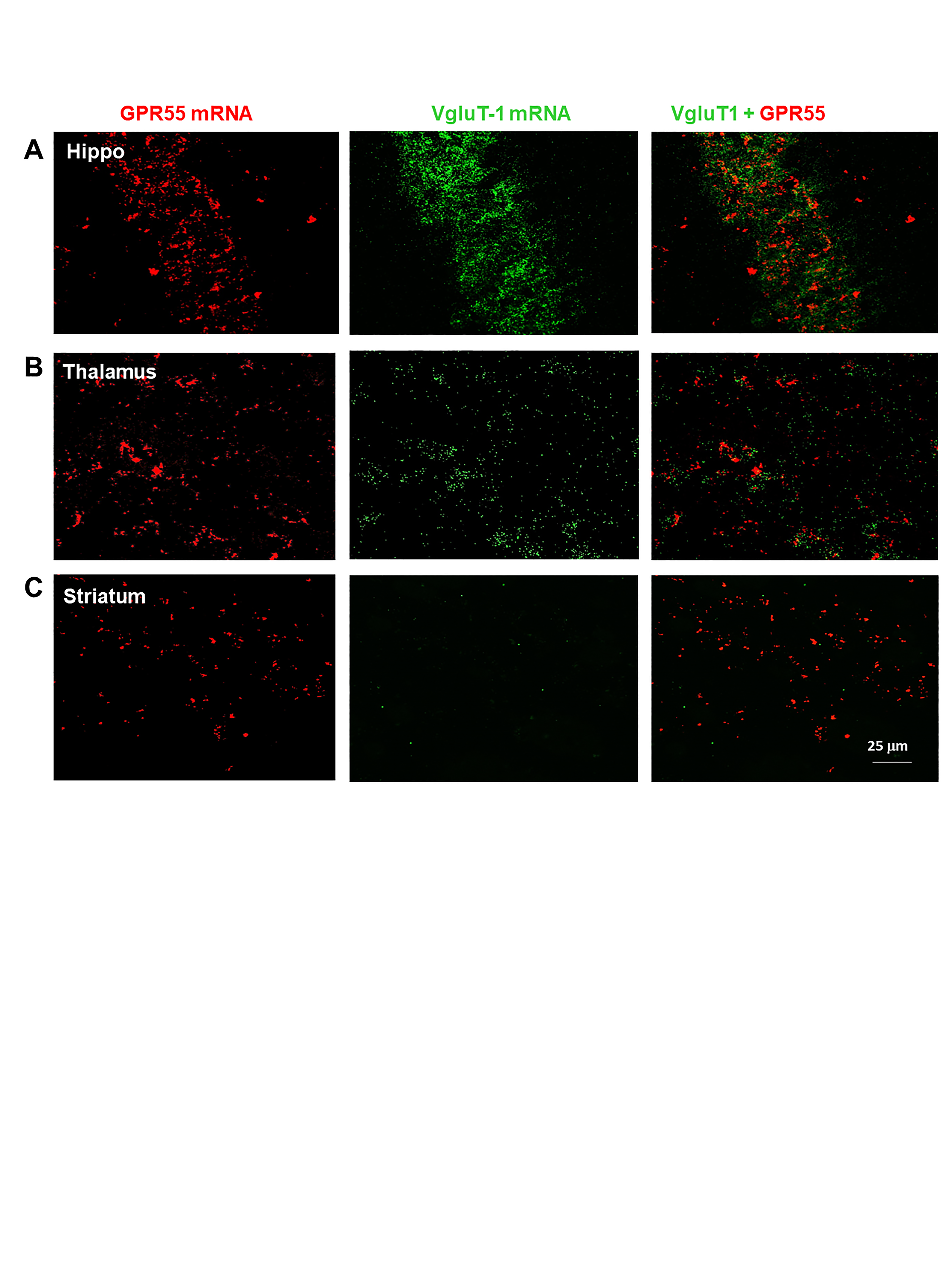

Supplement: Supplementary file 4 — Suppl. Fig. 3 - RNAscope - Hipp + [file 41398_2024_2820_MOESM4_ESM.tif]

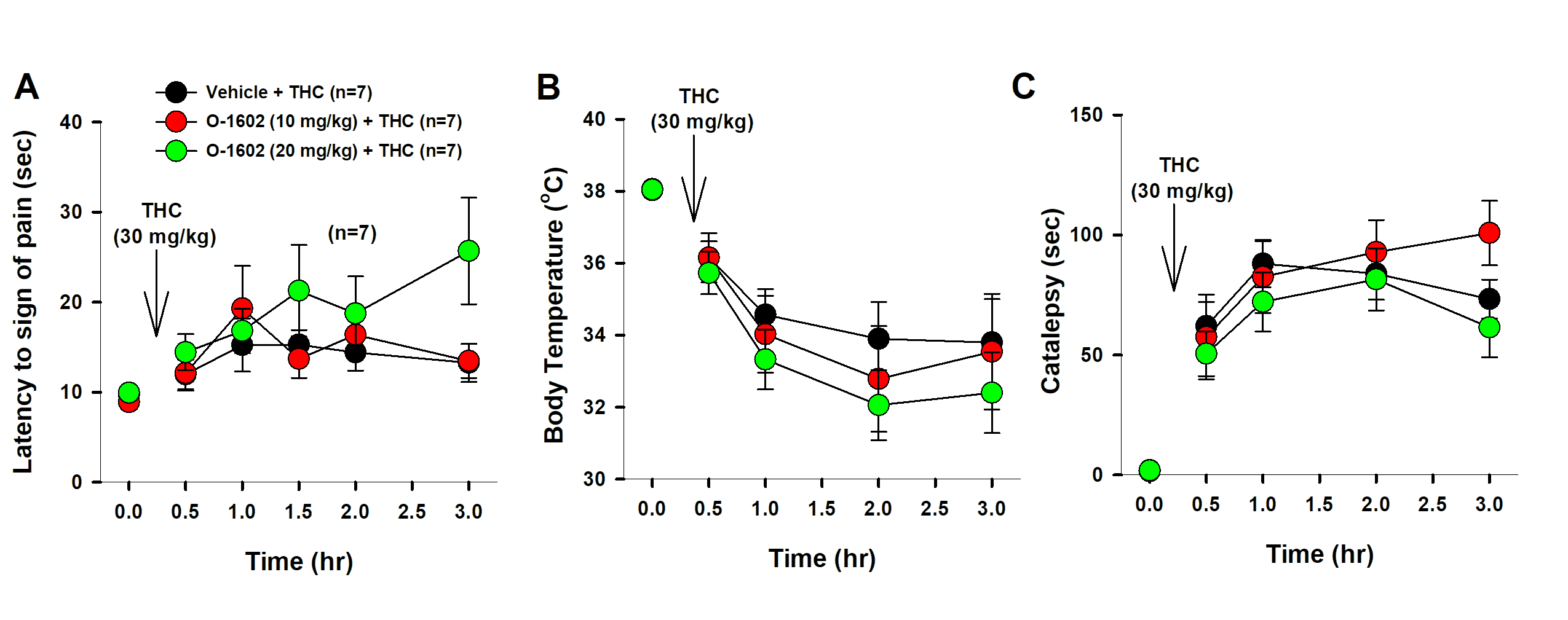

Supplement: Supplementary file 5 — Suppl. Fig. 4 - THC-Triad behavior [file 41398_2024_2820_MOESM5_ESM.tif]

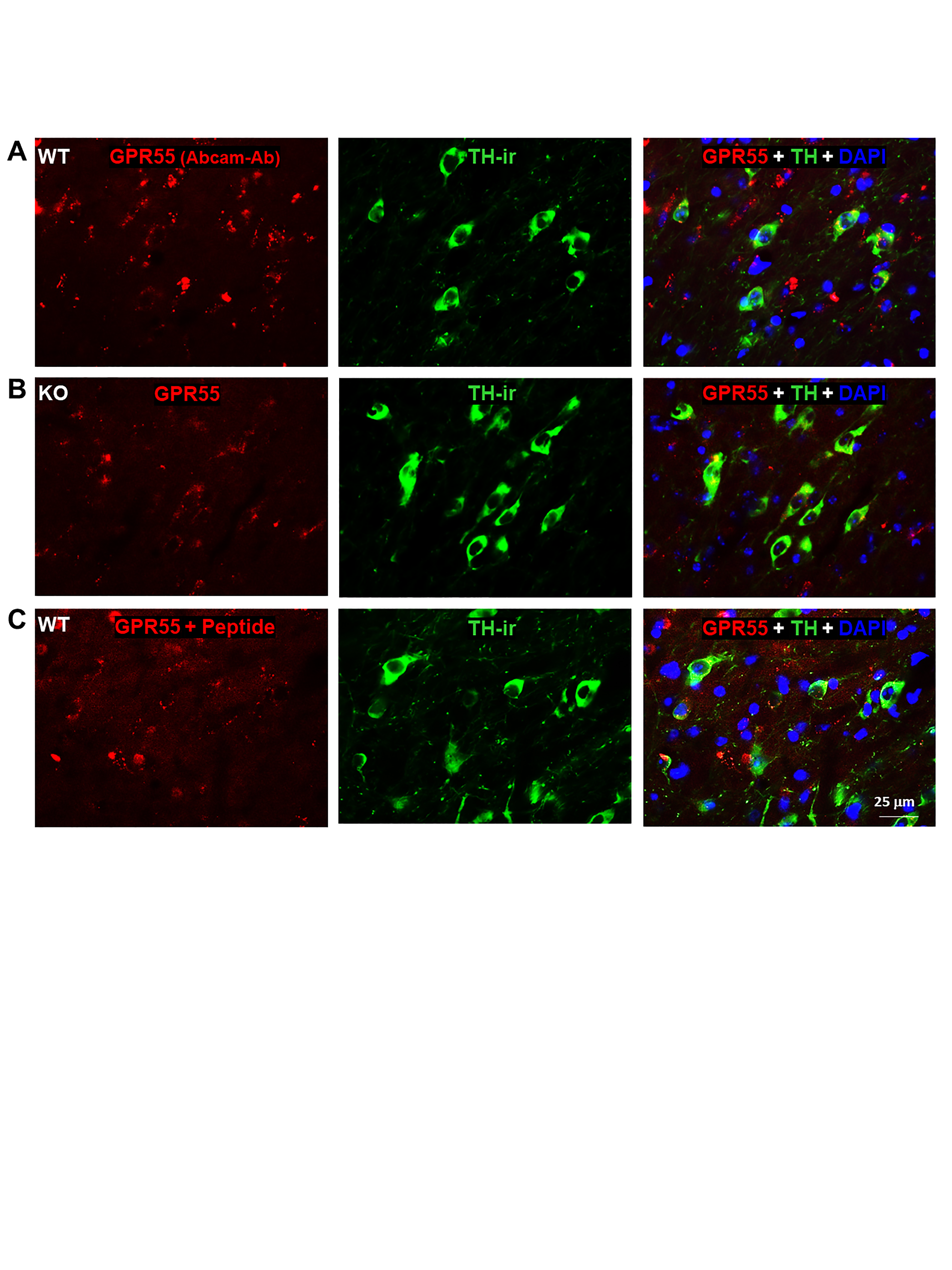

Supplement: Supplementary file 6 — Suppl. Fig. 5 - GPR55-IHC-Abcam Ab [file 41398_2024_2820_MOESM6_ESM.tif]

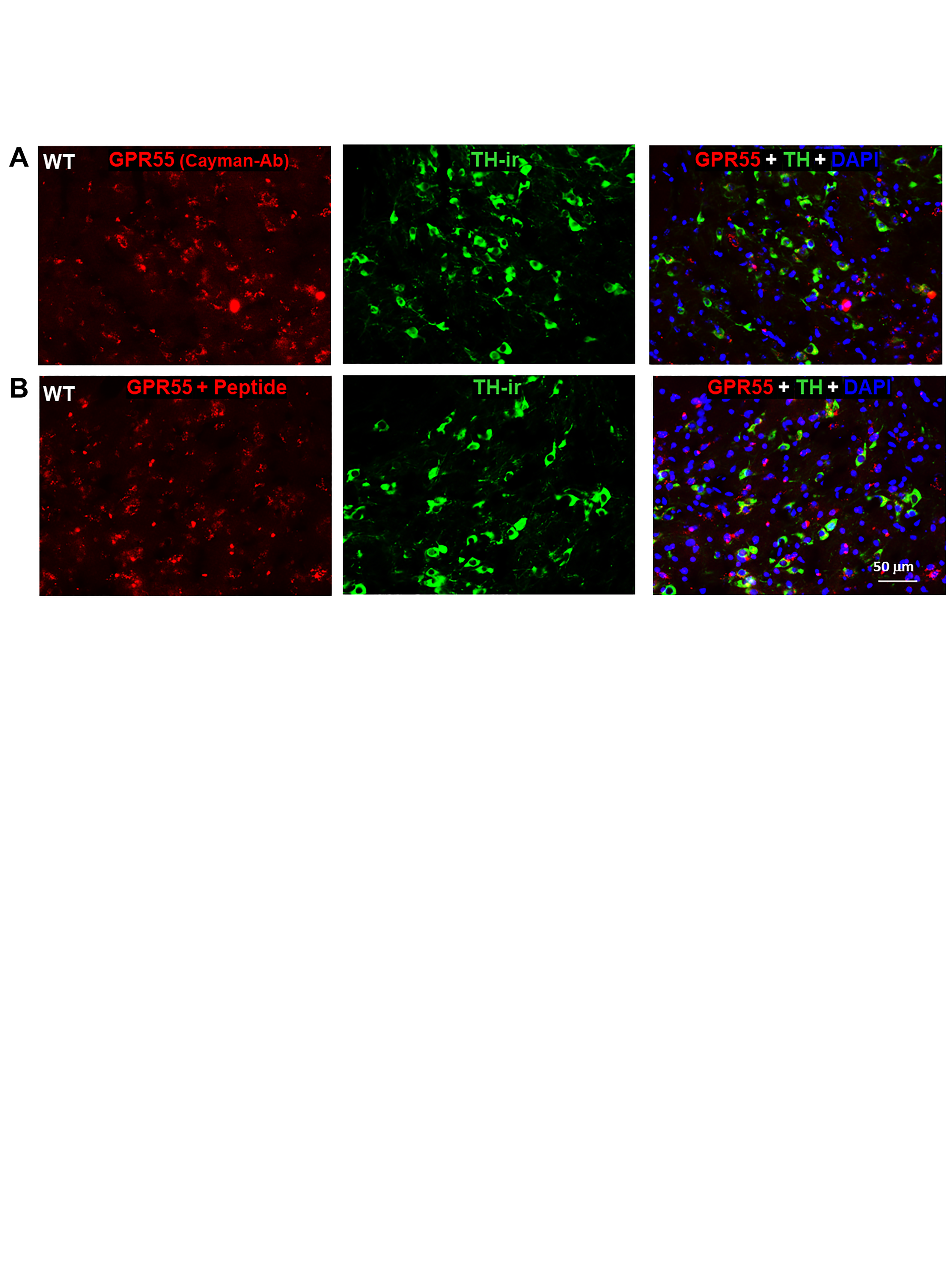

Supplement: Supplementary file 7 — Suppl. Fig. 6 - GPR55-IHC-Cayman Ab [file 41398_2024_2820_MOESM7_ESM.tif]

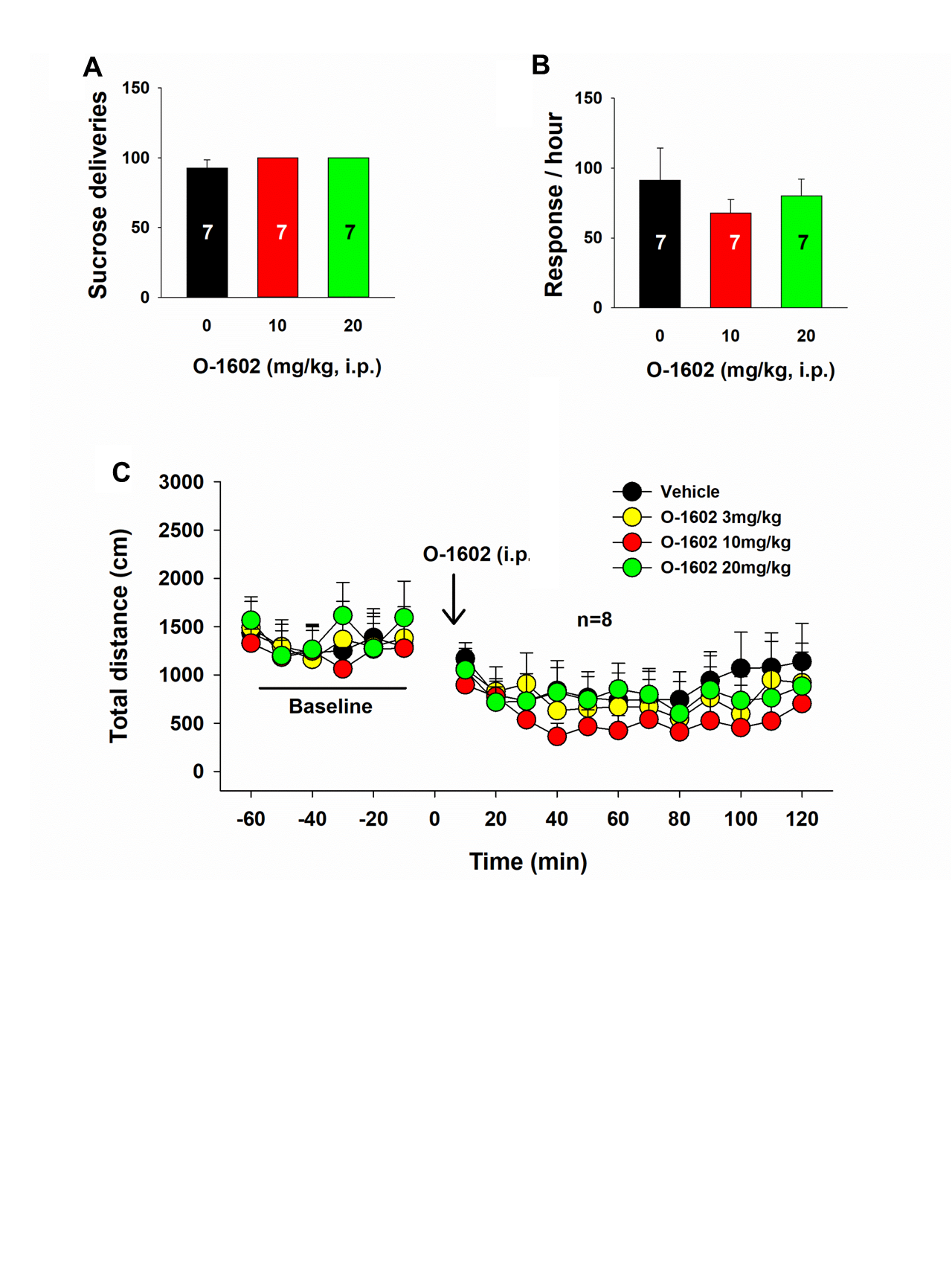

Supplement: Supplementary file 8 — Suppl. Fig. 7 - Sucrose SA + Locomotion [file 41398_2024_2820_MOESM8_ESM.tif]
